# Facile Chemical Access to Biologically Active Norcantharidin Derivatives from Biomass

**DOI:** 10.3390/molecules22122210

**Published:** 2017-12-12

**Authors:** Konstantin I. Galkin, Fedor A. Kucherov, Oleg N. Markov, Ksenia S. Egorova, Alexandra V. Posvyatenko, Valentine P. Ananikov

**Affiliations:** 1 Zelinsky Institute of Organic Chemistry, Russian Academy of Sciences, Leninsky Prospect 47, 119991 Moscow, Russia; glkn.ioc@gmail.com (K.I.G.); fkucherov@gmail.com (F.A.K.); markoff.o@yandex.ru (O.N.M.); egorova-ks@ioc.ac.ru (K.S.E.); 2 Institute of Gene Biology, Russian Academy of Sciences, Vavilova Str. 34/5, 119334 Moscow, Russia; sandra.posvjatenko@gmail.com; 3 Dmitry Rogachev National Medical Research Center of Pediatric Hematology, Oncology and Immunology, Ministry of Health of Russian Federation, Samory Mashela Str., 117198 Moscow, Russia

**Keywords:** biomass, 2,5-diformylfuran, reductive amination, norcantharidin, diastereoselectivity, Diels–Alder reaction

## Abstract

Reductive amination of 2,5-diformylfuran (DFF) was used to implement the transition from bio-derived 5-hydroxymethylfurfural (HMF) to pharmaceuticals. The synthesized bis(aminomethyl)furans were utilized as building blocks for the construction of new derivatives with structural cores of naturally occurring biologically active compounds. Using the one-pot procedure, which included the Diels–Alder reaction followed by hydrogenation of the double bond, bio-derived analogues of the anticancer drug norcantharidin were obtained. The cyclization process was diastereoselective, and resulted in the formation of tricyclic products with the endo configuration. Analysis of cytotoxycity for the resulting tricyclic amine-containing compounds showed an increase of anticancer activity as compared with the unsubstituted norcantharimide.

## 1. Introduction

Plant biomass is the largest renewable source of carbohydrates, with a mass of about 10^11^ tons of carbon per year [1]. To date, the most promising approach to the synthetic utilization of biomass involves the catalytic conversion of carbohydrates to molecular building blocks, which are often defined as platform chemicals [2,3]. 5-Hydroxymethylfurfural (HMF) is one of the most important platform chemicals, as it has many possibilities, especially in the fields of biofuels [4,5,6,7,8], biomaterials production [9,10,11,12], and fine chemical applications [13,14,15]. In contrast to several other classes of organic compounds, obtaining substituted amines from HMF is quite rare [13,14,15]. The most important applications are in the fields of medicine and material science. Indeed, the amine functional group is represented in many bioactive compounds, so the functionalization by an amine-containing fragment can improve the pharmacological properties of potential drugs. In addition, primary and secondary HMF-based amines can be used as monomers in the synthesis of bio-derived polyamides [9,16,17,18,19].

**Scheme 1:**
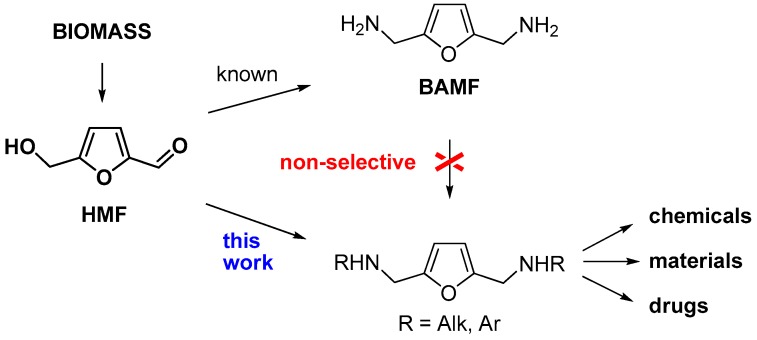
2,5-Bis(aminomethyl)furan (BAMF) synthesis from 5-hydroxymethylfurfural (HMF) and route to *N*-substituted BAMF derivatives.

The synthesis of 2,5-bis(aminomethyl)furan (BAMF), a useful diamine compound, from HMF is well known [16,20,21], but examples of the synthesis of *N*-substituted BAMF derivatives are rarely presented in the literature [22,23]. Possible ways to obtaining such derivatives are the alkylation of BAMF or the alkylation of primary amines by halogenated derivatives of HMF, especially by 5-(chloromethyl)furfural. Such transformations are not practical, since it is impossible to control the degree of alkylation and the formation of quaternary byproducts. Overall, these limitations lead to non-selective transformations (Scheme 1).

In the present work, we report the reductive amination of 2,5-diformylfuran (DFF) to connect biomass with pharmaceuticals containing an amino function (Scheme 1). For this purpose, we synthesized di- and tri-substituted BAMF derivatives as building blocks for further synthetic applications. The Diels–Alder protocol was used in order to obtain bio-derived analogues of the anticancer drug norcantharidin. Additionally, disubstituted amines were of considerable interest as monomers for the production of new biomaterials [19].

## 2. Results and Discussion

### 2.1. Synthesis of Norcantharidin Derivatives from HMF

**Scheme 2:**
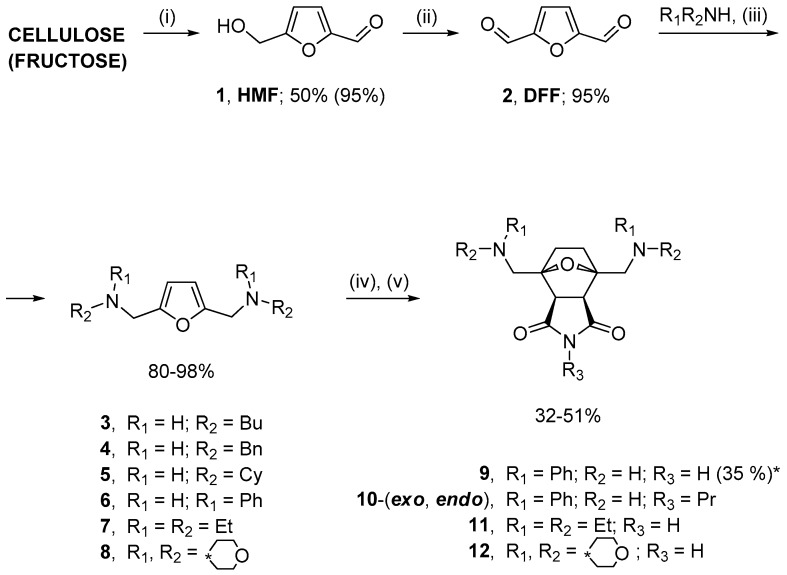
Reductive amination of 2,5-diformylfuran (DFF) for the synthesis of bio-based norcantharimides. (i) CrCl_3_·6H_2_O, [BMIM]Cl, 120 °C (H_2_SO_4_, [BMIM]Cl, 65 °C); (ii) [Pip’(O)][BF_4_], [BMIM]Cl; (iii) NaBH(OAc)_3_, CHCl_3_; (iv) maleimide, THF; (v) H_2_, 1 atm, 10% Pd/C, THF. *—yield using step-by-step procedure.

HMF was obtained from natural carbohydrates (cellulose or fructose), and was isolated in a pure crystalline form, as described previously (Scheme 2) [24,25]. DFF was synthesized by oxidation of HMF using a recyclable catalyst [Pip’(O)][BF_4_] (4-acetamido-2,2,6,6-tetramethyl-1-oxopiperidinium tetrafluoroborate) in ionic liquid media, according to the previously developed method [26]. In order to obtain amine-based building blocks, the reductive amination of DFF with mono- and disubstituted amines was implemented (Scheme 2). The reaction of DFF with two equivalents of amines, followed by the treatment with sodium triacetoxyborohydride resulted in amines **3**–**8**, which were obtained with high yields and very good purity of >95%.

The prepared 2,5-bis(aminomethyl)furan derivatives can be further modified at the amine or furan functional groups. The modification of substituents on the furan ring in HMF-based compounds has been well studied [13,14,15], whereas the chemistry of the furan ring is a challenging problem. For evaluation of the synthetic potential of the furan ring in the prepared amines, we synthesized norcantharimides **9**–**12**, which were bio-derived analogues of the anticancer drug norcantharidin. We used a modified one-pot protocol based on the Diels–Alder reaction of compounds **6**–**8** with alkenes, followed by hydrogenation of the formed double bond, as reported recently [27]. The Diels–Alder reactions were performed in THF media at room temperature and monitored by NMR. The second stage was performed by hydrogen addition over 10% Pd/C. Final products were isolated by column chromatography on silica with moderate yields and high purity.

Evaluation of the molecular structure of the formed tricyclic products was carried out using standard 1D-, 2D-NMR, and NOE experiments along with LC-MS data. In comparison with the previous results, the endo selectivity of the Diels–Alder reaction was observed for most of the isolated products. Introduction of the *N*-propyl moiety into maleimide led to the loss of diastereoselectivity of the cycloaddition, possibly due to the steric effects of the *N*-alkyl substituent, and the formation of the mixture of **10**-endo and **10**-exo products with a diastereomeric ratio of 4:1, respectively. Both diastereomers were isolated by column chromatography and characterized spectroscopically. The diastereomeric configuration was determined by ^1^H-NMR and NOE experiments. After considering the NMR data confirmed by the X-ray analysis of HMF-derived norcantharimide analogues obtained in the previous study [27], the spatial structure of the Diels–Alder adducts was confirmed (see the Supplementary Materials). Chemical shifts of the diastereomeric protons at the articulated position were characterized by a singlet at ~3.5–3.7 ppm in the ^1^H-NMR spectra, while the signals of the appropriate protons in the exo products (compounds norcantharimide [28] and **10**-exo) were observed in the stronger magnetic fields at ~2.9–3.1 ppm. Additional confirmation of the stereo configuration of compounds **10**-exo and **10**-endo was accomplished by nuclear Overhauser effect spectroscopy (NOESY) and analysis of the obtained spectral data (see the Supplementary Materials).

**Scheme 3:**
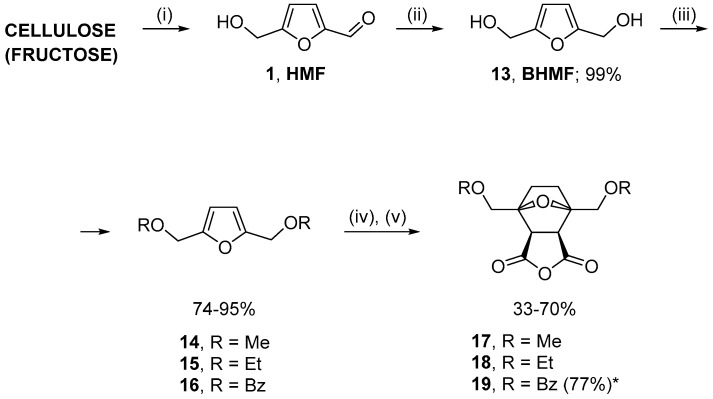
Preparation of norcantharidin analogues. (i) CrCl_3_·6H_2_O, [BMIM]Cl, 120 °C (H_2_SO_4_, [BMIM]Cl, 65 °C); (ii) NaBH_4_, water; (iii) alkylation or acylation; (iv) maleic anhydride, THF; (v) H_2_, 1 atm, 10% Pd/C, THF. *—yield using step-by-step procedure.

Norcantharidins and norcantharimides are synthetic analogues of the natural product cantharidin, which possesses anticancer activity and is of considerable practical interest [29,30,31,32,33,34]. For comparison of the cytotoxic activity of the prepared amine-containing tricyclic products, we prepared a number of their oxo analogues by the reaction of 2,5-(alkoxymethyl)furans **14**–**16** with maleic anhydride, followed by the hydrogenation reaction (Scheme 3). The initial furans were prepared by alkylation or acylation of BHMF using known methods (see the Section 3). The preparation of final products **17**–**19** was performed under the reaction conditions used for the synthesis of the furan–maleimide adducts, and endo selectivity of the cyclization reaction was observed.

The one-pot protocol for the synthesis of the tricyclic products was used because we were unable to isolate the intermediates of the Diels–Alder reaction due to their high polarity and the reversibility of the cycloaddition process. However, two unsaturated products were obtained using Et_2_O instead of THF, and were precipitated from the reaction mixture in pure form. The hydrogenation of these products in THF media under standard reaction conditions led to the formation of products **9** and **19,** with an overall yield that was higher than that when using the one-pot procedure (Scheme 3).

### 2.2. Biological Activity of Synthesized Compounds

**Figure 1:**
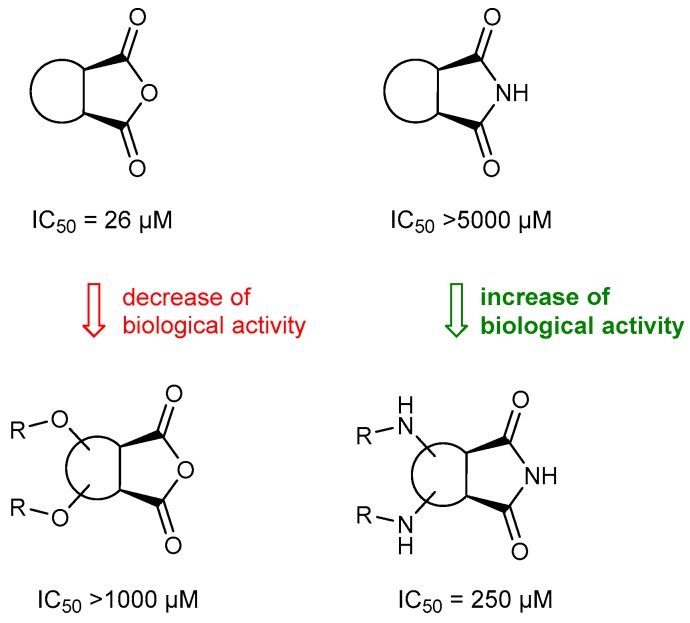
Increasing biological activity of cantharimide by introduction of an amino function (IC_50_ in HT-29 cells is given in µM).

Preliminary testing of the biological activity of selected substances was carried out (Figure 1). According to a cytotoxicity assay in human colorectal adenocarcinoma cell line HT-29, the activity of norcantharidin was in accordance with the published data [35]. Despite a recently reported improvement of anticancer activity of norcantharidin by introduction of *O*-benzoylated hydroxymethyl groups [29], isomeric product **19** and methylated analogue **17** did not show high cytotoxicity towards HT-29 cells. The cytotoxic activity of norcantharimide and its derivatives was low [36], but we found for the first time that it could be improved by introduction of the phenylaminomethyl moiety into the bicyclic core. The activity of aminomethylated norcantharimide **9** towards HT-29 cells was significantly higher than that of norcantharimide (see Figure 1 and the Supplementary Materials for details), but lower than the norcantharidin activity, indicating the necessity of further structural tuning to improve the biological activity.

## 3. Materials and Methods

Commercially available reagents and solvents were of analytical grade or were purified prior to use by standard methods. NMR spectra were recorded using a Bruker Fourier 300 HD, Bruker Avance III 400 and Bruker DRX-500 spectrometers (Fällanden, Switzerland) at the following frequencies: 300.1/400.1/500.1 MHz (^1^H), 75.5/100.6/125.8 MHz (^13^C). NMR chemical shifts were measured relative to residual protio solvent peaks. Mass spectrometric detection was performed on a high resolution Bruker maXis instrument equipped with an electrospray ionization (ESI) ion source. Chromatographic separations were performed on silica gel (Kieselgel, 230–400 mesh, Merck Schuchardt, Darmstadt, Germany) with analytical grade solvents. Analytical TLC was performed on Merck silica gel plates with a QF-254 indicator. Visualization was accomplished with UV light or using KMnO_4_ in aqueous NaOH or (NH_4_)_6_Mo_7_O_24_ in ethanolic H_2_SO_4_, and the TLC was heated until the development of color. Norcantharidin and norcantharimide were obtained from commercial sources.

### 3.1. Synthesis of Substrates

*5-Hydroxymethylfurfural* (HMF, **1**) was prepared from cellulose or fructose with 50% (45% after recrystallization) or 95% (90% after recrystallization) yields, respectively [24].

*2,5-Diformylfuran* (DFF, **2**) was prepared from **1** with 95% yield [26].

*2,5-Bis(hydroxymethyl)furan* (BHMF, **13**) was prepared from **1** with 99% yield [37].

*2,5-Bis(methoxymethyl)furan* (**14**) was prepared from **13** with 74% yield [38].

*2,5-Bis(ethoxymethyl)furan* (**15**) was prepared from **13** with 76% yield [27].

*Furan-2,5-diylbis(methylene)dibenzoate* (**16**) was prepared from **13** with 95% yield [39]. ^1^H-NMR (500 MHz, CDCl_3_) *δ* = 8.06 (d, *J* = 7.8 Hz, 4H), 7.55 (t, *J* = 7.1 Hz, 2H), 7.42 (t, *J* = 7.5 Hz, 4H), 6.49 (s, 2H), 5.31 (s, 4H). ^13^C-NMR (126 MHz, CDCl_3_) *δ* 166.3, 150.4, 133.2, 129.9, 128.5, 111.9, 58.6. HRMS (ESI) Calcd. for C_20_H_16_O_5_ [M + Na]: 359.0890. Found 359.0875.

### 3.2. General Method for Synthesis of 2,5-Bis(aminomethyl)furans

A solution of 2,5-diformylfuran **2** (0.5 g, 4 mmol) and the corresponding amine (8.4 mmol) in chloroform (15 mL) was stirred for 20 min, and sodium triacetoxyborohydride (3.4 g, 16 mmol) was added portion-wise. The reaction mixture was stirred overnight, poured into a saturated solution of sodium bicarbonate, and extracted with chloroform (3 × 7 mL). The combined organic phases were washed with water, dried with sodium sulfate, evaporated, and dried under high vacuum. Target compounds **3**–**8** were obtained as pale yellow oils.

*2,5-Bis(N-butylaminomethyl)furan* (**3**). Yield 0.89 g, 93%. ^1^H-NMR (500 MHz, CDCl_3_) *δ* = 6.05 (s, 2H), 3.71 (s, 4H), 2.58 (t, *J* = 7.2 Hz, 4H), 1.45 (dt, *J* = 14.7, 7.3 Hz, 4H), 1.30 (m, 4H), 0.88 (t, *J* = 7.3 Hz, 6H). ^13^C-NMR (126 MHz, CDCl_3_) *δ* = 153.4, 107.4, 49.0, 46.5, 32.2, 20.5, 14.1. HRMS (ESI) Calcd. for C_14_H_26_N_2_O [M + H]: 239.2118. Found 239.2129.

*2,5-Bis(N-benzylaminomethyl)furan* (**4**). Yield 0.99 g, 80%. ^1^H-NMR (500 MHz, CDCl_3_) *δ* = 7.34 (d, *J* = 4.4 Hz, 8H), 7.27 (dt, *J* = 6.8, 4.4 Hz, 2H), 6.13 (s, 2H), 3.80 (d, *J* = 13.5 Hz, 8H), 1.73 (s, 2H). ^13^C-NMR (126 MHz, CDCl_3_) *δ* = 153.2, 140.0, 128.5, 128.3, 127.1, 107.7, 52.9, 45.6. HRMS (ESI) Calcd. for C_20_H_22_N_2_O [M + H]: 307.1805. Found 307.1815.

*2,5-Bis(N-cyclohexylaminomethyl)furan* (**5**). Yield 1.10 g, 95%. ^1^H-NMR (500 MHz, CDCl_3_) *δ* = 6.04 (s, 2H), 3.75 (s, 4H), 2.42 (m, 2H), 1.85 (d, *J* = 10.6 Hz, 4H), 1.71 (d, *J* = 12.9 Hz, 4H), 1.59 (d, *J* = 12.0 Hz, 2H), 1.40 (br. s, 2H), 1.30–1.00 (m, 10H). ^13^C-NMR (126 MHz, CDCl_3_) *δ* = 153.6, 107.2, 55.9, 43.6, 33.5, 26.3, 25.1. HRMS (ESI) Calcd. for C_18_H_30_N_2_O [M + H]: 291.2431. Found 291.2442.

*2,5-Bis(N-phenylaminomethyl)furan* (**6**). Yield 1.02 g, 92%. ^1^H-NMR (500 MHz, CDCl_3_) *δ* = 7.20 (t, *J* = 7.9 Hz, 4H), 6.76 (t, *J* = 7.3 Hz, 2H), 6.69 (d, *J* = 7.7 Hz, 4H), 6.17 (s, 2H), 4.29 (s, 4H). ^13^C-NMR (126 MHz, CDCl_3_) *δ* = 152.1, 147.5, 129.4, 118.4, 113.5, 108.1, 41.8. HRMS (ESI) Calcd. for C_18_H_18_N_2_O [M + Na]: 301.1311. Found 301.1322.

*2,5-Bis(N,N-diethylaminomethyl)furan* (**7**). Yield 760 mg, 80% [27]. ^1^H-NMR (500 MHz, CDCl_3_) *δ* = 6.10 (s, 2H), 3.67 (s, 4H), 2.52 (q, 8H, *J* = 7.2 Hz), 1.08 (t, 12H, *J* = 7.2 Hz). ^13^C-NMR (126 MHz, CDCl_3_) *δ* = 151.4, 109.1, 48.6, 46.9, 12.0. HRMS (ESI) Calcd. for C_14_H_26_N_2_O [M + H]: 239.2118. Found 239.2118.

*2,5-Bis(morpholinomethyl)furan* (**8**). Yield 1.04 g, 98% [27]. ^1^H-NMR (500 MHz, CDCl_3_) *δ* = 6.13 (s, 2H), 3.70 (m, 8H), 3.51 (s, 4H), 2.45 (m, 8H). ^13^C-NMR (126 MHz, CDCl_3_) *δ* = 150.9, 109.7, 66.8, 55.3, 53.2. HRMS (ESI) Calcd. for C_14_H_22_N_2_O_3_ [M + H]: 267.1703. Found 267.1703.

### 3.3. General Method for One-Pot Synthesis of Norcantharimide Derivatives

To a solution of 2,5-bis(aminomethyl)furans **6**–**8** (0.6 mmol) in 2 mL of THF, maleimide (0.12 g, 1.2 mmol, 2 eq.) was added. The reaction mixture was stirred for 24 h at 24 °C. Approximately 20 mg of 10% Pd/C was added, and the reaction mixture was placed under a hydrogen atmosphere (1 atm) for 8 h at 24 °C. The catalyst was filtered off and washed thoroughly with hot THF (3 × 4 mL). The filtrate was evaporated under reduced pressure. The residue was purified by flash chromatography (ethyl acetate as eluent). Target compounds **10**–**12** were obtained as pale-yellow oils. Diastereomeric products **10**-endo and **10**-exo were formed in a ratio of 4:1 and separated using column chromatography.

*endo-4,7-Bis((phenylamino)methyl)-2-propylhexahydro-1H-4,7-epoxyisoindole-1,3(2H)-dione(endo-4,7-bis-(phenylamino)-2-propyl-norcantharimide)* (**10**-endo). Yield 133 mg, 53%. ^1^H-NMR (500 MHz, CDCl_3_) δ = 7.22 (t, J = 7.8 Hz, 8H), 6.77 (t, J = 7.4 Hz, 12H), 4.37 (br. s, 2H), 3.70 (d, J = 13.3 Hz, 2H), 3.59 (d, J = 13.3 Hz, 2H), 3.53–3.41 (m, 4H), 1.89 (d, J = 7.6 Hz, 2H), 1.76 (d, J = 7.7 Hz, 2H), 1.63 (m, 1H), 0.97 (t, J = 7.4 Hz, 1H). ^13^C-NMR (126 MHz, CDCl_3_) δ = 176.2, 148.0, 129.4, 118.1, 113.2, 88.6, 53.4, 47.3, 40.7, 30.0, 21.1, 11.5. HRMS (ESI) Calcd. for C_25_H_29_N_3_O_3_ [M + Na]: 442.2101. Found 442.2114.

exo-4,7-Bis((phenylamino)methyl)-2-propylhexahydro-1H-4,7-epoxyisoindole-1,3(2H)-dione(exo-4,7-bis(phe-nylamino)-2-propyl-norcantharimide) (**10**-exo). Yield 33 mg, 13%. ^1^H-NMR (400 MHz, CDCl_3_) δ = 7.19 (t, J = 7.9 Hz, 4H), 6.72 (dd, J = 12.5, 7.6 Hz, 6H), 4.21 (s, 2H), 3.69 (q, J = 14.0 Hz, 4H), 3.49 (t, J = 7.1 Hz, 2H), 3.10 (s, 2H), 2.06 (d, J = 7.2 Hz, 2H), 1.76 (d, J = 7.2 Hz, 2H), 1.61 (m, 2H), 0.88 (t, J = 7.4 Hz, 3H). ^13^C-NMR (126 MHz, CDCl_3_) δ = 176.1, 148.2, 129.4, 117.9, 113.4, 88.2, 52.1, 45.4, 40.7, 34.2, 21.1, 11.3. HRMS (ESI) Calcd. for C_25_H_29_N_3_O_3_ [M + Na]: 442.2101. Found 442.2111.

*endo-4,7-Bis((diethylamino)methyl)hexahydro-1H-4,7-epoxyisoindole-1,3(2H)-dione(endo-4,7-bis(diethyla-mino)norcantharimide)* (**11**). Yield 103 mg, 51% [27]. ^1^H-NMR (500 MHz, CD_3_OD) *δ* = 3.66 (s, 2H), 2.99 (m, 4H), 2.76–2.89 (m, 8H), 1.76–1.85 (m, 4H), 1.12 (t, 12H, *J* = 7.1 Hz). ^13^C-NMR (126 MHz, CD_3_OD) *δ* = 177.8, 88.6, 54.5, 53.6, 48.4, 29.1, 10.3.

*endo-4,7-Bis(morpholinomethyl)hexahydro-1H-4,7-epoxyisoindole-1,3(2H)-dione(endo-4,7-bis(morpholino-methyl)norcantharimide)* (**12**). Yield 93 mg, 42% [27]. ^1^H-NMR (500 MHz, CD_3_OD) *δ* = 3.58–3.65 (m, 10H), 2.72 (m, 4H), 2.48–2.66 (m, 8H), 1.69–1.83 (m, 4H). ^13^C-NMR (126 MHz, CD_3_OD) *δ* = 178.2, 89.1, 66.7, 59.8, 54.6, 53.7, 28.9.

### 3.4. General Method for One-Pot Synthesis of Norcantharidin Derivatives

To a solution of compounds **14**–**16** (1 mmol) in 2 mL of THF, maleic anhydride (196 mg, 2 mmol, 2 eq.) was added, and the reaction mixture was stirred for 24 h at 24 °C. Approximately 30 mg of 10% Pd/C was added, and the reaction mixture was placed under a hydrogen atmosphere (1 atm) for 12 h at 24 °C. The catalyst was filtered off and washed thoroughly with hot THF (3 × 4 mL). The filtrate was evaporated under reduced pressure, and the resulting mixture was treated by column chromatography. Target compounds **17**–**19** were obtained as white solids or light yellow oils.

*endo-4,7-Bis(methoxymethyl)hexahydro-4,7-epoxyisobenzofuran-1,3-dione(endo-4,7-bis(methoxymethyl)-norcantharidin)* (**17**). Yield 43 mg, 17% (33% based on reacted substrate). ^1^H-NMR (500 MHz, CDCl_3_) *δ* = 3.86 (d, *J* = 10.6 Hz, 2H), 3.76 (d, *J* = 10.6 Hz, 2H), 3.42 (s, 6H), 3.31 (s, 2H), 2.06 (d, *J* = 7.4 Hz, 2H), 1.71 (d, *J* = 7.5 Hz, 2H). ^13^C-NMR (126 MHz, CDCl_3_) *δ* = 169.3, 88.1, 70.8, 59.7, 52.5, 32.0. HRMS (ESI) Calcd. for C_12_H_16_O_6_ [M + Na]: 279.0839. Found 279.0836.

*endo-4,7-Bis(ethoxymethyl)hexahydro-4,7-epoxyisobenzofuran-1,3-dione(endo-4,7-bis(ethoxymethyl)nor-cantharidin)* (**18**). Yield 77 mg, 27% (59% based on reacted substrate). ^1^H-NMR (500 MHz, CDCl_3_) *δ* = 3.92 (d, *J* = 10.6 Hz, 2H), 3.81 (d, *J* = 10.6 Hz, 2H), 3.61 (m, 4H), 3.31 (s, 2H), 2.08 (q, *J* = 3.5 Hz, 2H), 1.73 (q, *J* = 3.4 Hz, 2H), 1.22 (t, *J* = 7.0 Hz, 6H). ^13^C-NMR (126 MHz, CDCl_3_) *δ* = 169.4, 88.3, 68.8, 67.5, 52.6, 32.2, 15.1. HRMS (ESI) Calcd. for C_14_H_20_O_6_ [M + H]: 307.1152. Found 307.1148.

endo-1,3-Dioxohexahydro-4,7-epoxyisobenzofuran-4,7-diyl)bis(methylene)dibenzoate(endo-4,7-bis((O-ben-zoyl)-oxymethyl)-norcantharidin) (**19**). Yield 112 mg, 26% (70% based on reacted substrate). ^1^H-NMR (500 MHz, DMSO-d_6_) δ = 7.97 (d, J = 7.4 Hz, 4H), 7.68 (t, J = 7.4 Hz, 2H), 7.54 (t, J = 7.6 Hz, 4H), 4.72 (m, 4H), 3.80 (s, 2H), 2.01 (s, 4H). ^13^C-NMR (126 MHz, DMSO-d_6_) δ = 170.6, 165.1, 133.6, 129.3, 128.8, 86.6, 62.4, 52.9, 31.2. HRMS (ESI) Calcd. for C_24_H_20_O_8_ [M + H]: 459.1050. Found 459.1040.

### 3.5. Synthesis of Compounds ***9*** and ***19*** Using Step-by-Step Procedure

#### 3.5.1. Preparation of Compound **9**

To a solution of compound **6** (100 mg, 0.36 mmol) in 0.2 mL of Et_2_O, maleimide (105 mg, 1.1 mmol, 3 eq.) was added, and the reaction mixture was stirred for 24 h at 24 °C. The precipitate that formed was filtered, washed with cold Et_2_O, and dried in vacuo. Compound **9a** was obtained as white crystals (49 mg, 36%). Approximately 10 mg of 10% Pd/C was added to a solution of compound **9a** (49 mg, 0.13 mmol) in THF (1 mL), and the reaction mixture was placed under a hydrogen atmosphere (1 atm) for 8 h at 24 °C. The catalyst was filtered off and washed thoroughly with hot THF (3 × 4 mL). The filtrate was evaporated under reduced pressure. The residue was purified by flash chromatography (ethyl acetate as eluent). Compound **9** was obtained as a pale yellow oil (47 mg, 35% over two steps).

*endo-4,7-Bis((phenylamino)methyl)-3a,4,7,7a-tetrahydro-1H-4,7-epoxyisoindole-1,3(2H)-dione* (**9a**). ^1^H·NMR (500 MHz, Acetone-*d*_6_) *δ* = 9.80 (s, 1H), 7.14 (t, *J* = 7.8 Hz, 4H), 6.78 (d, *J* = 7.9 Hz, 4H), 6.64 (t, *J* = 7.3 Hz, 2H), 6.52 (s, 2H), 5.00 (s, 2H), 3.80 (qd, *J* = 13.7, 6.0 Hz, 4H), 3.67 (s, 2H). ^13^C-NMR (126 MHz, Acetone-*d*_6_) *δ* 176.8, 149.8, 137.5, 129.9, 117.9, 113.7, 92.1, 52.2, 45.8. HRMS (ESI) Calcd. for C_22_H_11_N_3_O_3_ [M + H]: 376.1656. Found 376.1648.

*endo-4,7-Bis((phenylamino)methyl)hexahydro-1H-4,7-epoxyisoindole-1,3(2H)-dione(endo-4,7-bis(phenylami-no)norcantharimide)* (**9**). ^1^H-NMR (500 MHz, CDCl_3_) *δ* = 8.26 (s, 1H), 7.21 (t, *J* = 7.6 Hz, 4H), 6.75 (t, *J* = 8.9 Hz, 6H), 4.19 (s, 2H), 3.68 (d, *J* = 13.6 Hz, 2H), 3.58 (d, *J* = 10.3 Hz, 4H), 1.93 (s, 4H). ^13^C-NMR (126 MHz, CDCl_3_) *δ* = 176.1, 148.1, 129.5, 118.2, 113.2, 88.9, 54.6, 47.1, 30.1. HRMS (ESI) Calcd. for C_22_H_23_N_3_O_3_ [M + H]: 378.1812. Found 378.1818.

#### 3.5.2. Preparation of Compound **19**

To a solution of compound **16** (100 mg, 0.3 mmol) in 2 mL of Et_2_O, maleic anhydride (87 mg, 0.9 mmol, 3 eq.) was added, and the reaction mixture was stirred for 24 h at 24 °C. The precipitate that formed was filtered, washed with cold Et_2_O, and dried in vacuo. Compound **19a** was obtained as white crystals (110 mg, 84%). Approximately 10 mg of 10% Pd/C was added to a solution of compound **19a** (110 mg, 0.25 mmol) in THF (1 mL), and the reaction mixture was placed under a hydrogen atmosphere (1 atm) for 8 h at 24 °C. The catalyst was filtered off and washed thoroughly with hot THF (3 × 4 mL). The filtrate was evaporated under reduced pressure. The residue was purified by flash chromatography (ethyl acetate as eluent). Compound **19** was obtained as a pale yellow oil (100 mg, 77% over two steps).

*endo-1,3-Dioxo-1,3,3a,7a-tetrahydro-4,7-epoxyisobenzofuran-4,7-diylbis(methylene)dibenzoate* (**19a**). ^1^H-NMR (500 MHz, Acetone-*d*_6_) *δ* = 8.02 (d, *J* = 7.6 Hz, 4H), 7.65 (t, *J* = 7.3 Hz, 2H), 7.50 (t, *J* = 7.6 Hz, 4H), 6.84 (s, 2H), 5.13 (d, *J* = 12.6 Hz, 2H), 4.85 (d, *J* = 12.6 Hz, 2H), 3.90 (s, 2H). ^13^C-NMR (75 MHz, Acetone-*d*_6_) *δ* 169.8, 166.2, 139.2, 134.2, 130.7, 130.4, 129.5, 91.4, 62.3, 53.8. HRMS (ESI) Calcd. for C_24_H_18_O_8_ [M + NH_4_]: 452.1340. Found 452.1342.

### 3.6. Preliminary Studies of Cytotoxicity of Selected Substances

#### 3.6.1. Cell Culture

The human colorectal adenocarcinoma cell line HT-29 (The Russian Cell Culture Collection (RCCC), the Institute of Cytology RAS, St. Petersburg, Russia) was cultured in clear plastic tissue culture-treated dishes or multiple-well plates (Corning Inc., Corning, NY, USA) in a HeraCell 150 incubator (Thermo Electron Corp., Waltham, MA, USA) at 37 °C, 95% humidity, and 5% CO_2_. The DMEM (Dulbecco’s modified Eagle’s medium)/F-12 (1:1) medium with 2.5 mM L-glutamine and 1.5 mM HEPES (4-(2-hydroxyethyl)-1-piperazineethanesulfonic acid) (HyClone, Logan, UT, USA) was used. The medium was supplemented with 10% fetal bovine serum (HyClone, Logan, UT, USA), 100 units·mL^−1^ penicillin (OAO Sintez, Kurgan, Russia), and 100 μg*mL^−1^ streptomycin (OAO Biokhimik, Saransk, Russia).

#### 3.6.2. MTS Assay

The MTS assay was used to evaluate the cytotoxicity of the target compounds, as previously described [40]. Before the test, cells were placed into 96-well flat-bottomed plates, with 10,000 cells per well. The utmost wells were filled with 200 μL of phosphate-buffered saline (HyClone, Logan, UT, USA). Cells were cultivated until reaching a 70% monolayer, and then were incubated for 48 h or 72 h with the target compounds in concentrations ranging from 1 μM to 5000 μM, depending on the compound, with 10 points in total. The same substance concentrations were applied to an empty plate to allow an adjustment for the substance influence on optical density. All of the test points were measured in triplicate. Triton-100 (1%, Sigma-Aldrich, St. Louis, MO, USA) in the culture medium was used as a positive control, and the medium was used as a negative control. After incubation, 20 μL of the MTS (3-(4,5-dimethylthiazol-2-yl)-5-(3-carboxymethoxyphenyl)-2-(4-sulfophenyl)-2*H*-tetrazolium) reagent (CellTiter 96^®^ AQueous One Solution Cell Proliferation Assay, Promega, Fitchburg, WI, USA) were added into each well, and the plates were incubated for additional 4 h. Optical density was measured at 492 nm and 650 nm using an Original Multiskan EX (Lab Systems, Midland, ON, Canada), and the values obtained at 650 nm were subtracted from those obtained at 492 nm to exclude background absorption.

## 4. Conclusions

In summary, we have developed the reductive amination of DFF in order to obtain new amine-containing bio-derived building blocks **3**–**6** and norcantharidin derivatives **9**, **10**, and **17**–**19**, which can be used in the production of drugs and materials. The prepared derivatives extend applications of HMF as a platform chemical in organic synthesis, particularly connecting biomass processing with amine-containing building blocks. An efficient one-pot procedure was implemented, and the Diels–Alder reaction of substituted BAMF derivatives with maleimide and maleic anhydride was carried out with high endo selectivity; the influence of substituents in maleimide on diastereoselectivity of the cycloaddition was observed. It was shown that the introduction of amines in the side-chains resulted in the enhanced bioactivity of cantharimide.

## Supplementary Materials

The spectra of synthesized compounds and Table S1: Cytotoxicity of selected substances towards HT-29 cells, are available online.
