# Supplementary material for: Facile Chemical Access to Biologically Active Norcantharidin Derivatives from Biomass
Source: Molecules. 2017 Dec 12;22(12):2210. doi: 10.3390/molecules22122210 (PMC6149738; doi:10.3390/molecules22122210)
Supplement: Supplementary file 1 [file molecules-22-02210-s001.pdf]

Electronic supplementary information

for the article

Facile chemical access to biologically active norcantharidin derivatives from biomass

Konstantin I. Galkin, Fedor A. Kucherov, Oleg N. Markov, Ksenia S. Egorova, Alexandra V. Posvyatenko and Valentine P. Ananikov*

Zelinsky Institute of Organic Chemistry, Russian Academy of Sciences, Leninsky prospect 47, 119991 Moscow, Russia. E-mail: val@ioc.ac.ru, http://AnanikovLab.ru

Table of Contents

NMR and MS spectra of synthesized compounds..2

Preliminary studies of cytotoxicity of selected substances 21

NMR and MS spectra of synthesized compounds

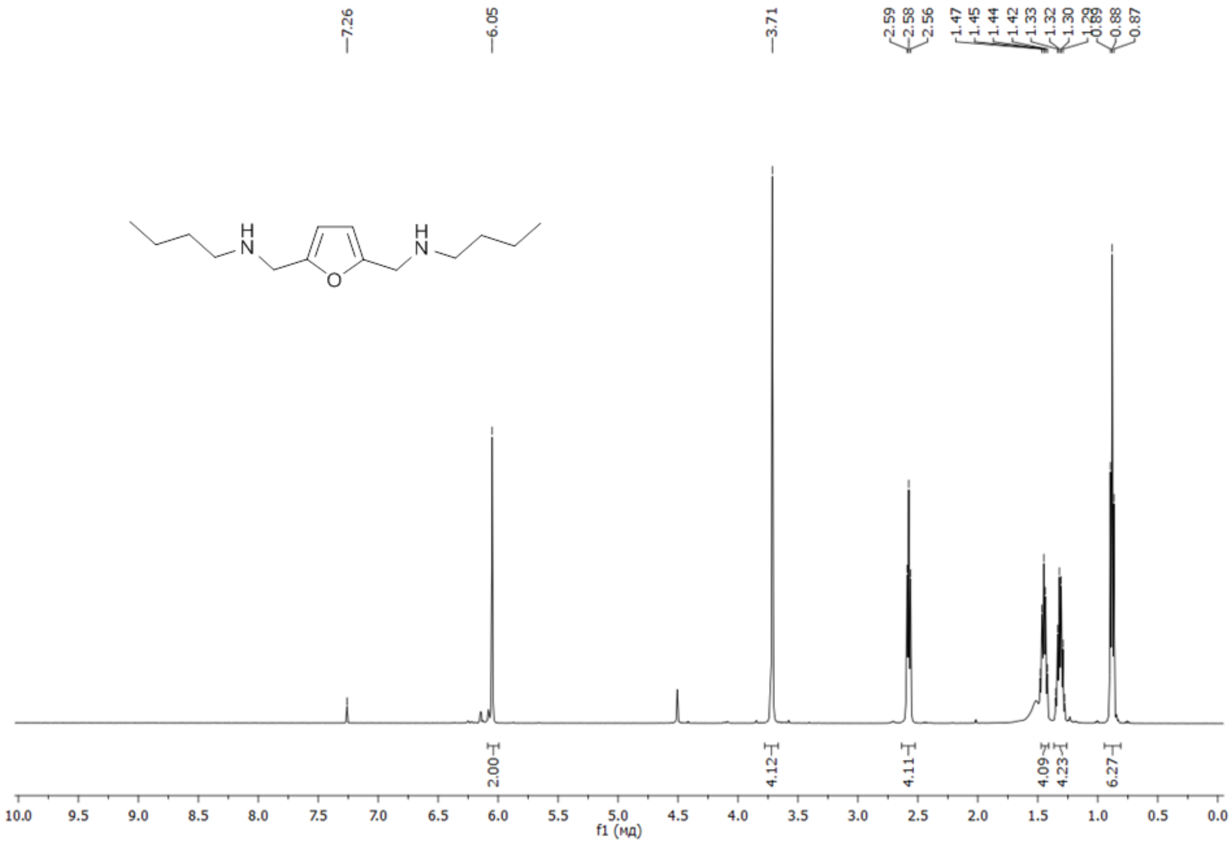

Fig. S1. **^{1}H NMR spectrum of compound 3 ($CDCl_3$, 298 K, 500 MHz).**

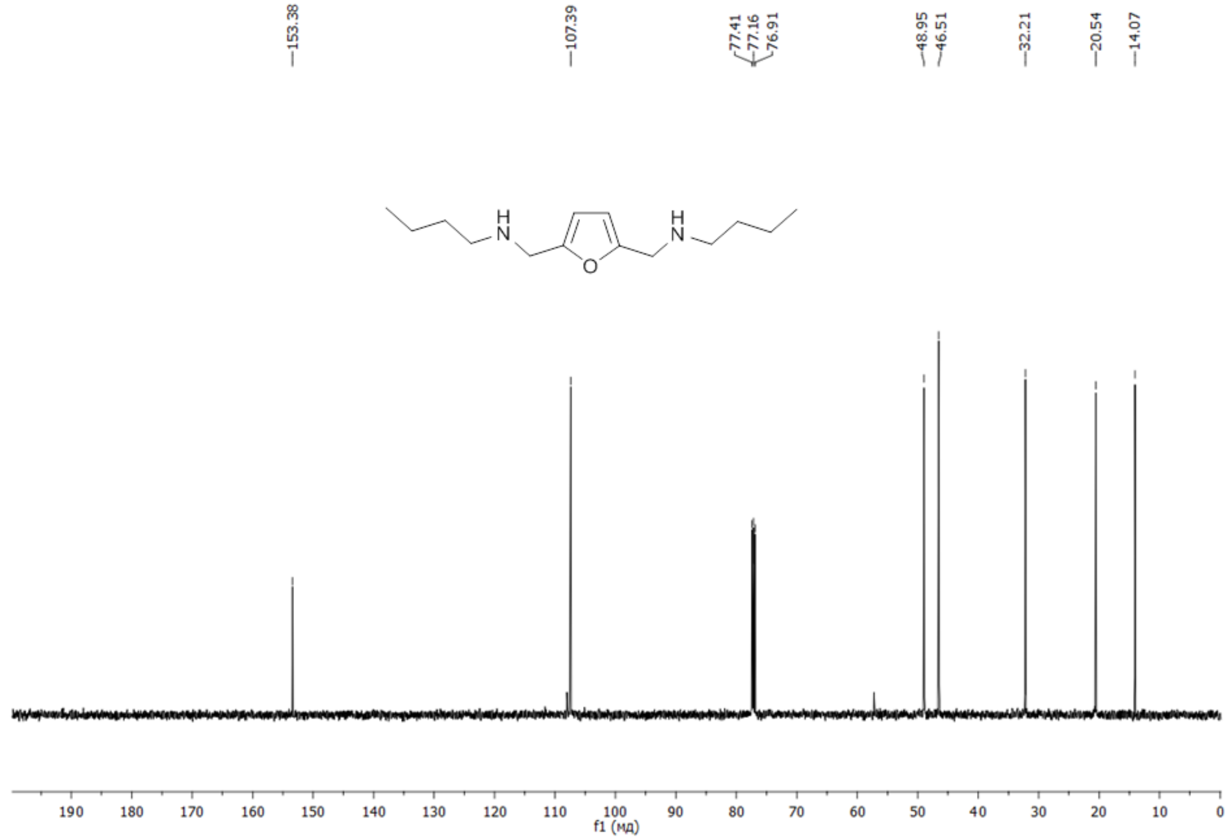

Fig. S2. **^{13}C NMR spectrum of compound 3 ($CDCl_3$, 298 K, 126 MHz).**

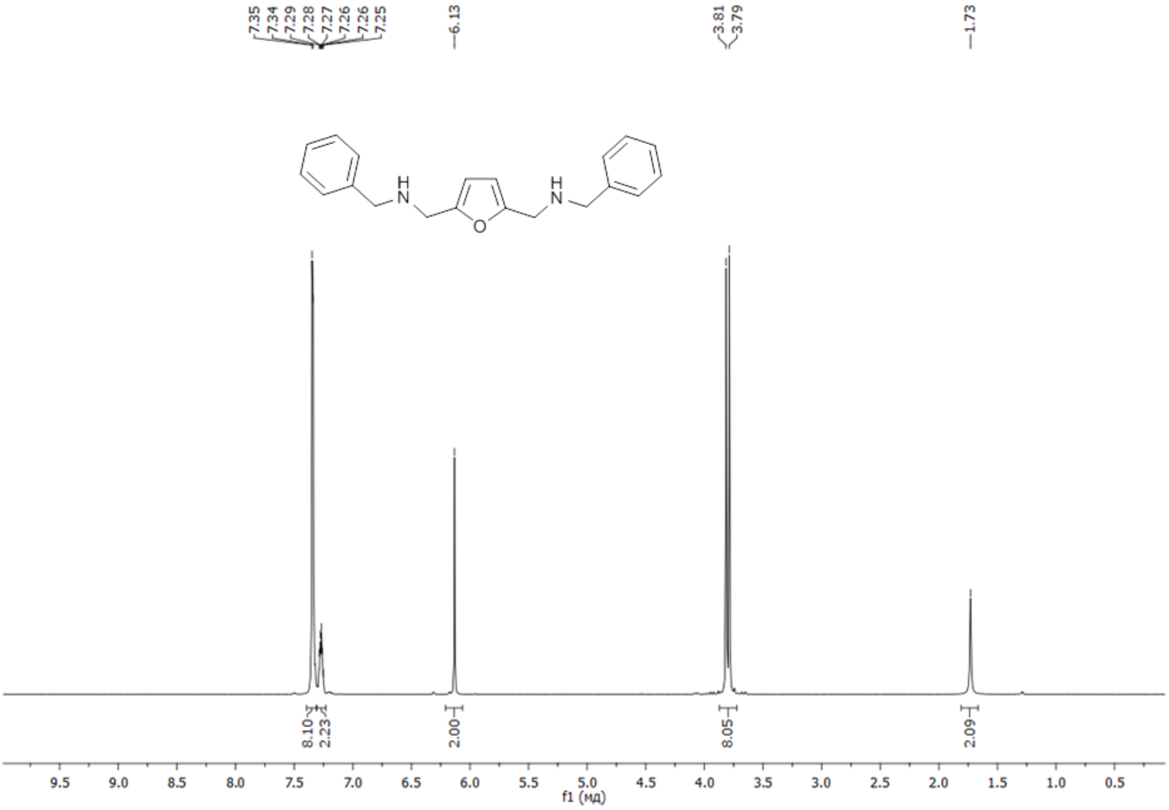

Fig. S3. [1]**H NMR spectrum of compound 4 ($CDCl_3$, 298 K, 500 MHz).**

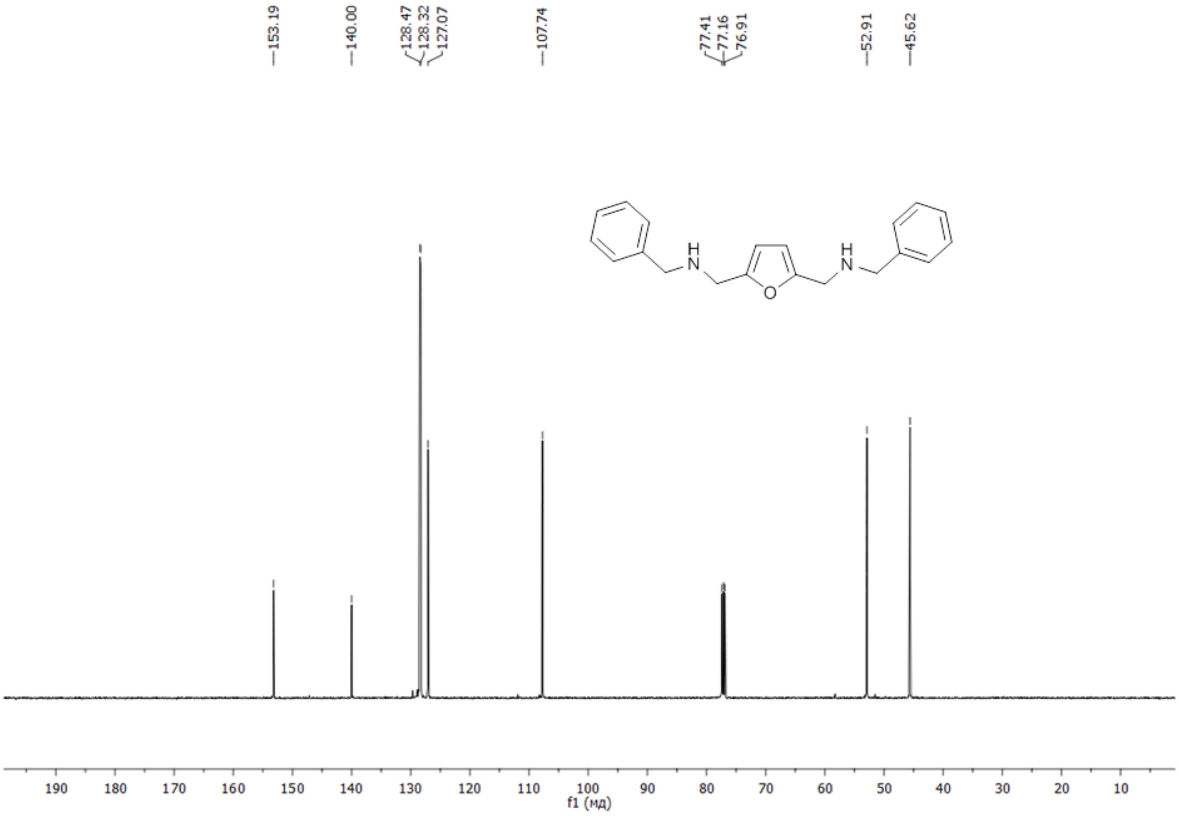

Fig. S4. [13]**C NMR spectrum of compound 4 ($CDCl_3$, 298 K, 126 MHz).**

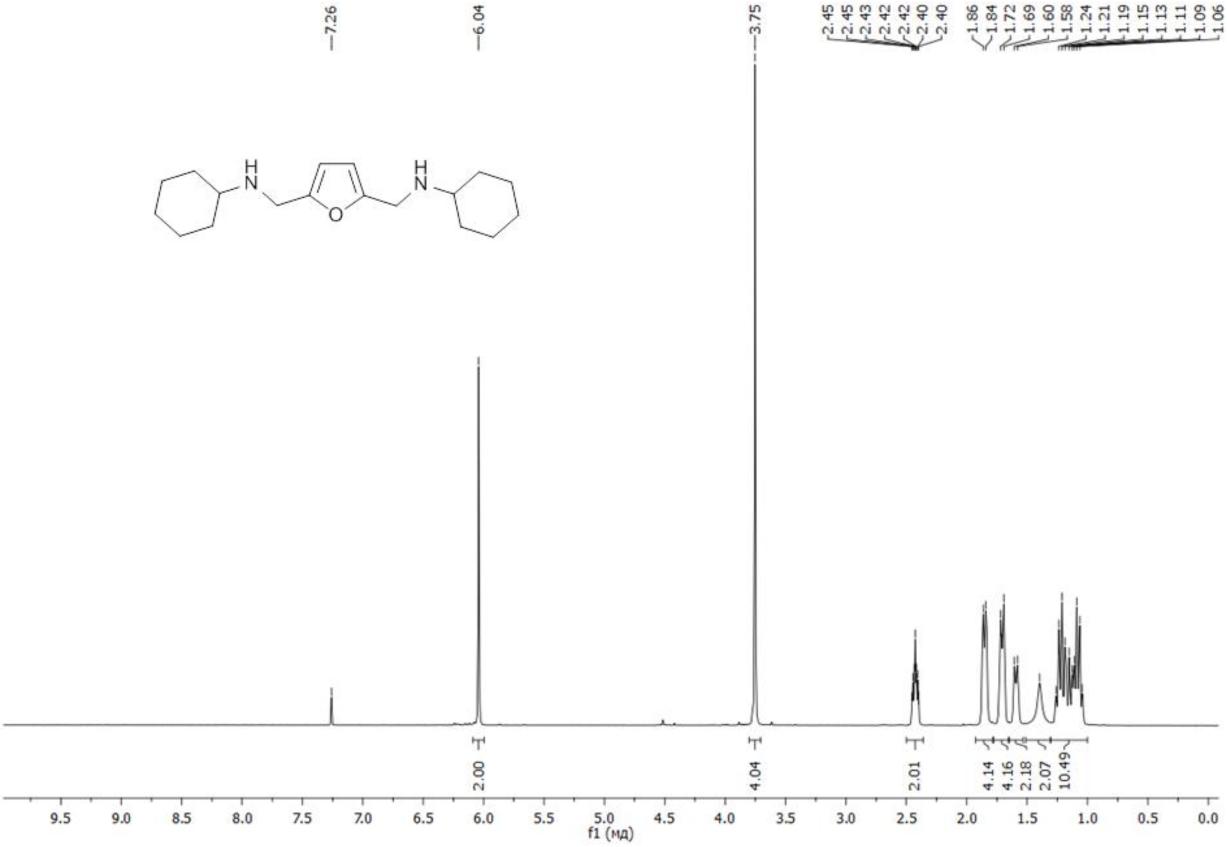

Fig. S5. ^{1}H NMR spectrum of compound 5 ($CDCl_3$, 298 K, 500 MHz).

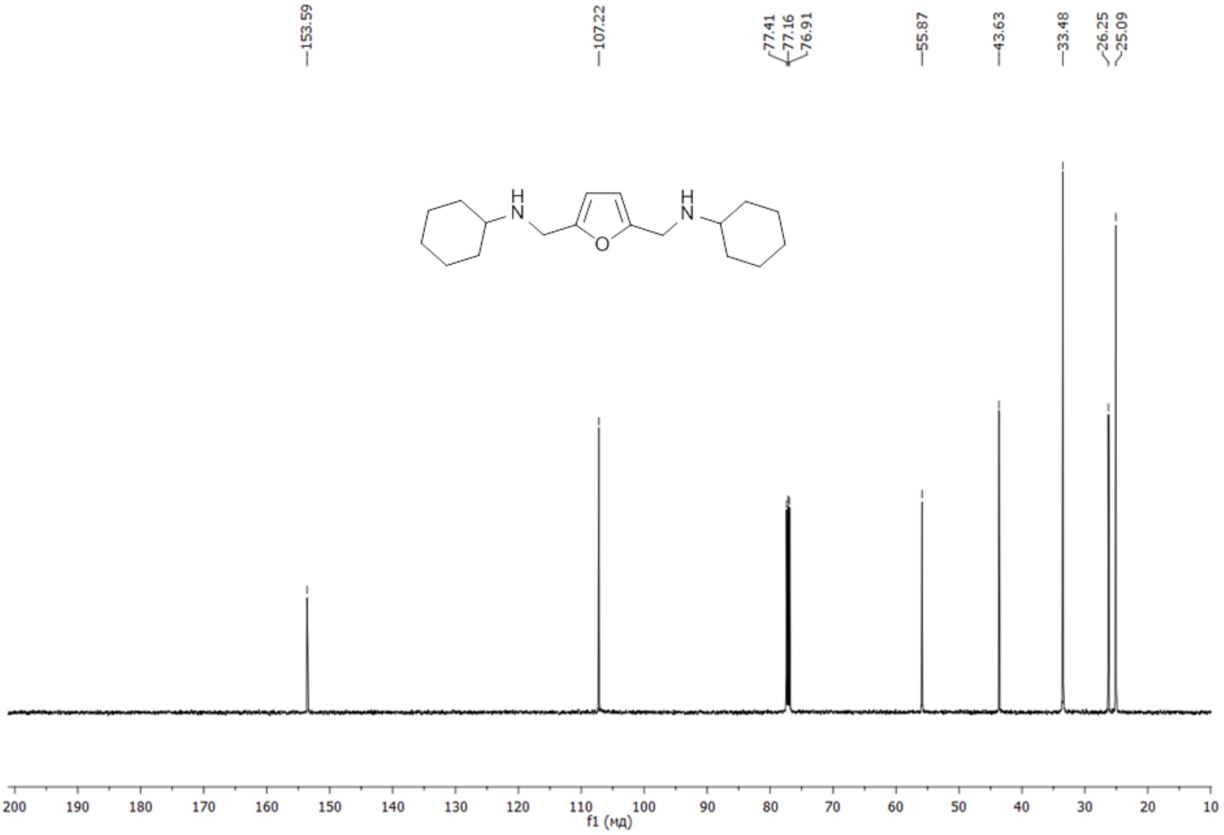

Fig. S6. ^{13}C NMR spectrum of compound 5 ($CDCl_3$, 298 K, 126 MHz).

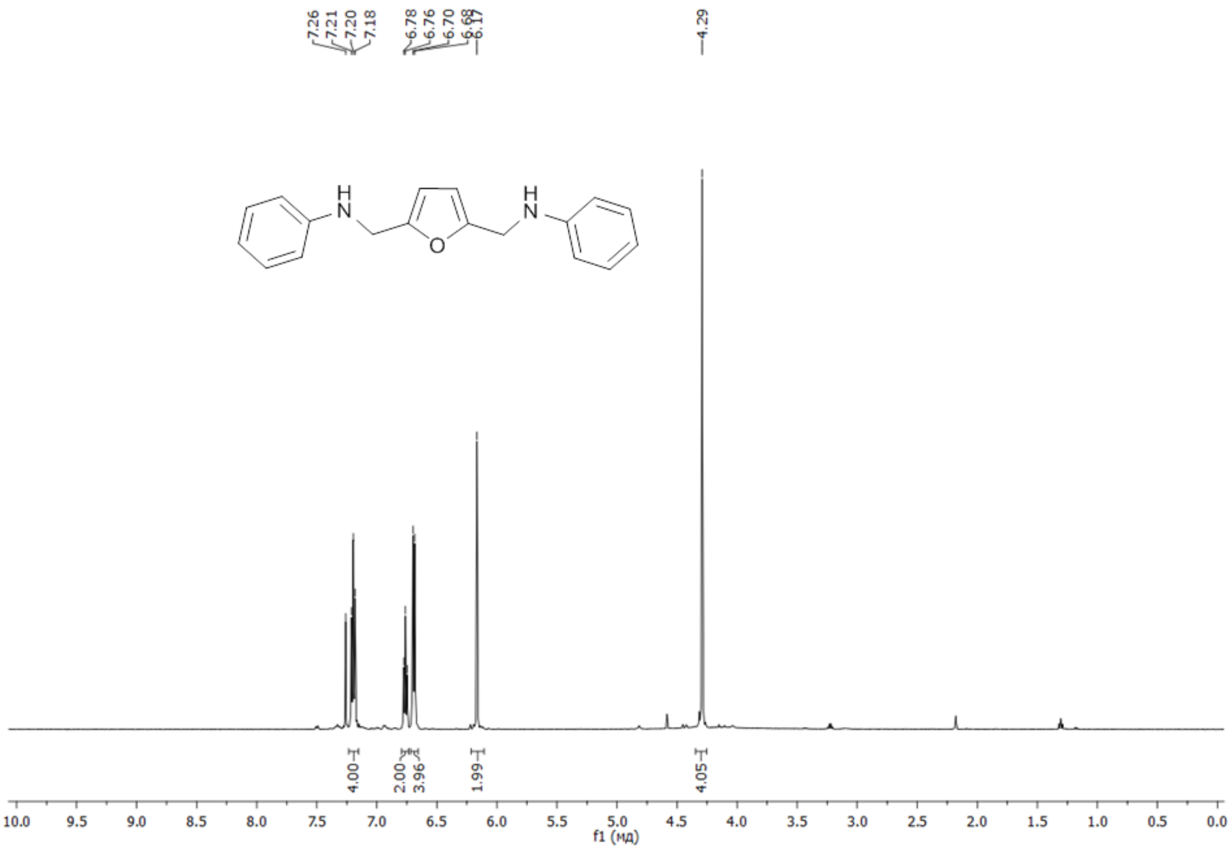

Fig. S7. ^{1}**H NMR spectrum of compound 6 (CDCl$_3$, 298 K, 500 MHz).**

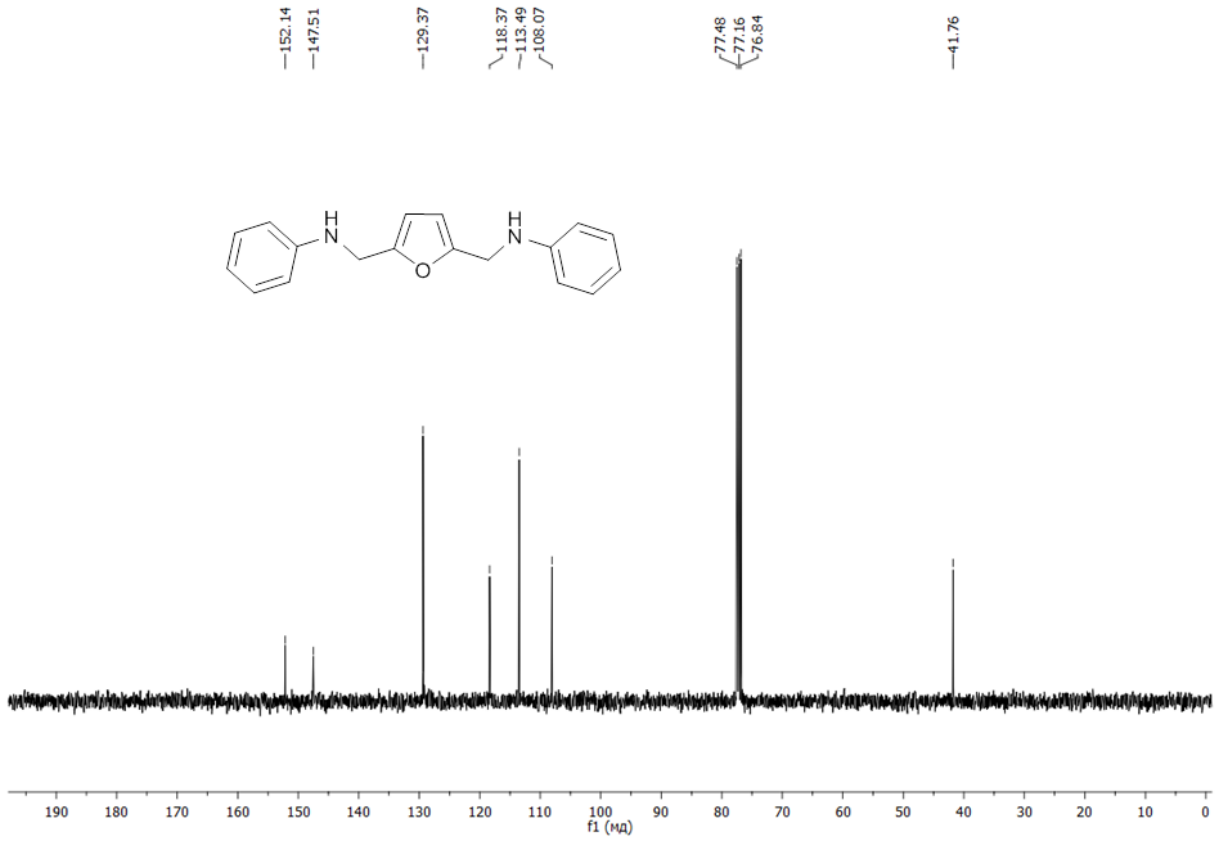

Fig. S8. ^{13}**C NMR spectrum of compound 6 (CDCl$_3$, 298 K, 126 MHz).**

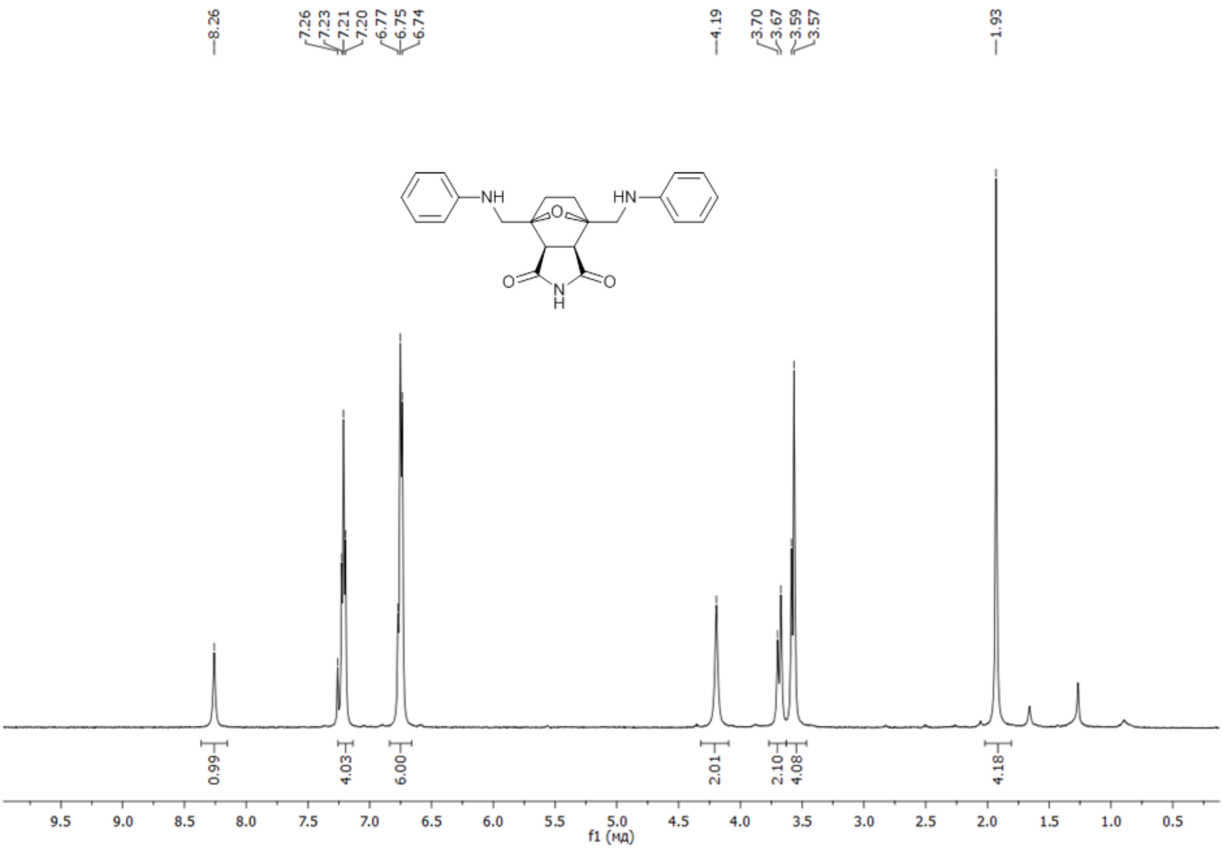

Fig. S9. ^{1}**H NMR spectrum of compound 9 (**$CDCl_3$**, 298 K, 500 MHz).**

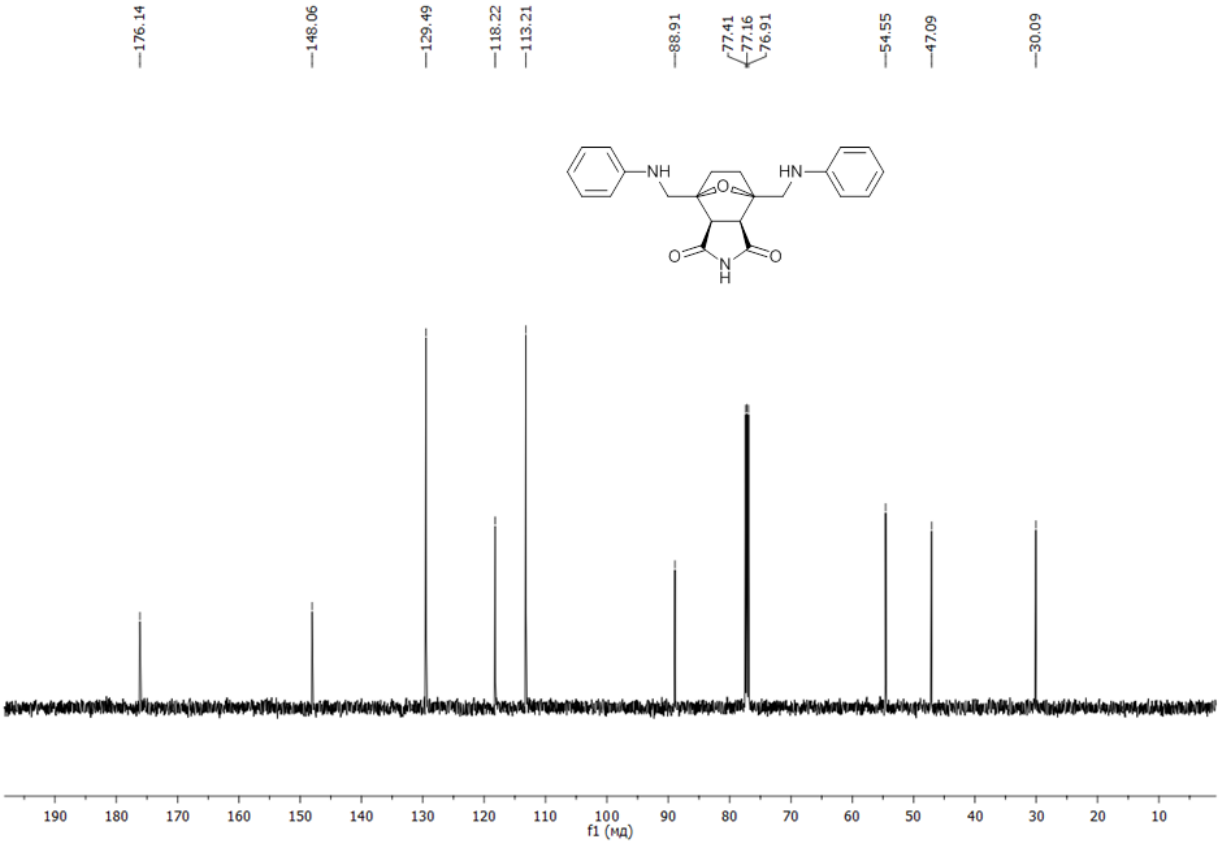

Fig. S10. ^{13}**C NMR spectrum of compound 9 (**$CDCl_3$**, 298 K, 126 MHz).**

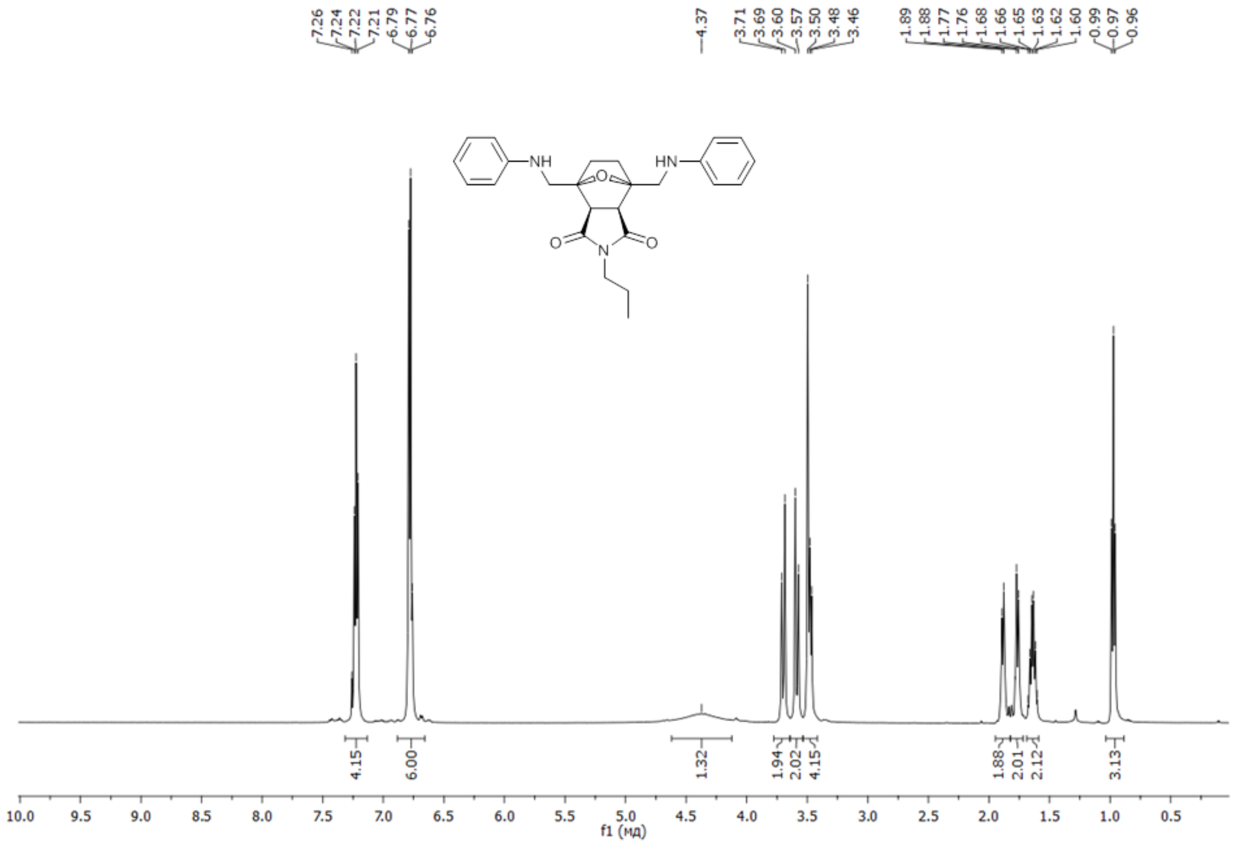

Fig. S11. **^{1}H NMR spectrum of compound 10-endo ($CDCl_3$, 298 K, 500 MHz).**

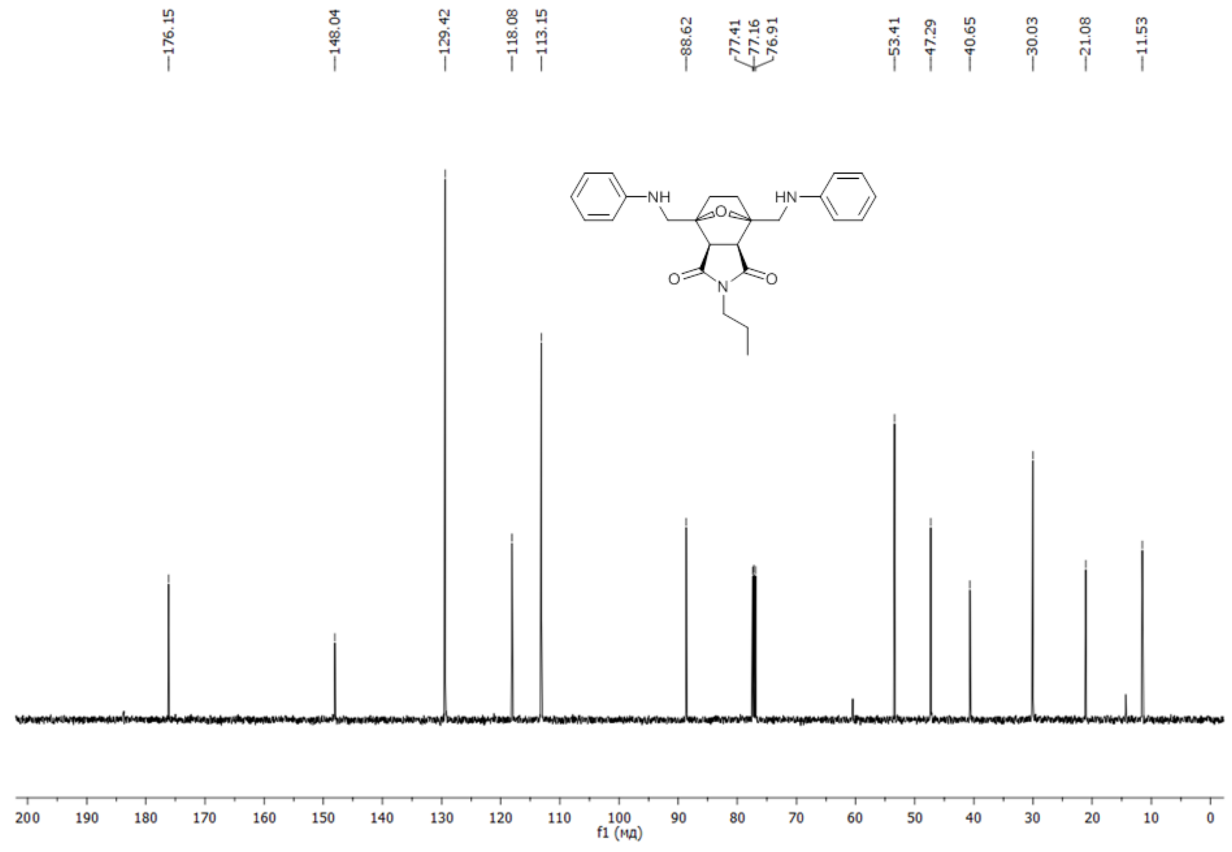

Fig. S12. **^{13}C NMR spectrum of compound 10-endo ($CDCl_3$, 298 K, 126 MHz).**

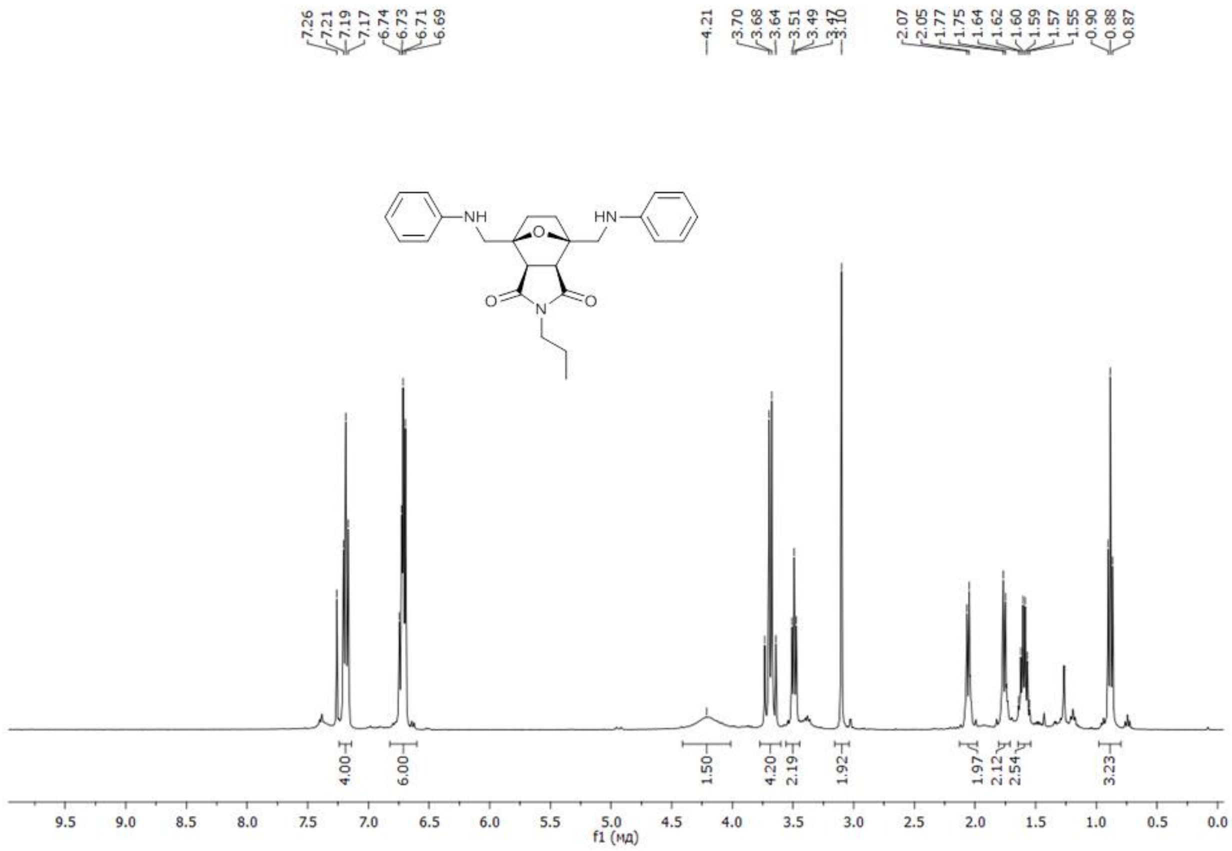

Fig. S13. ^{1}H NMR spectrum of compound 10-exo ($CDCl_3$, 298 K, 400 MHz).

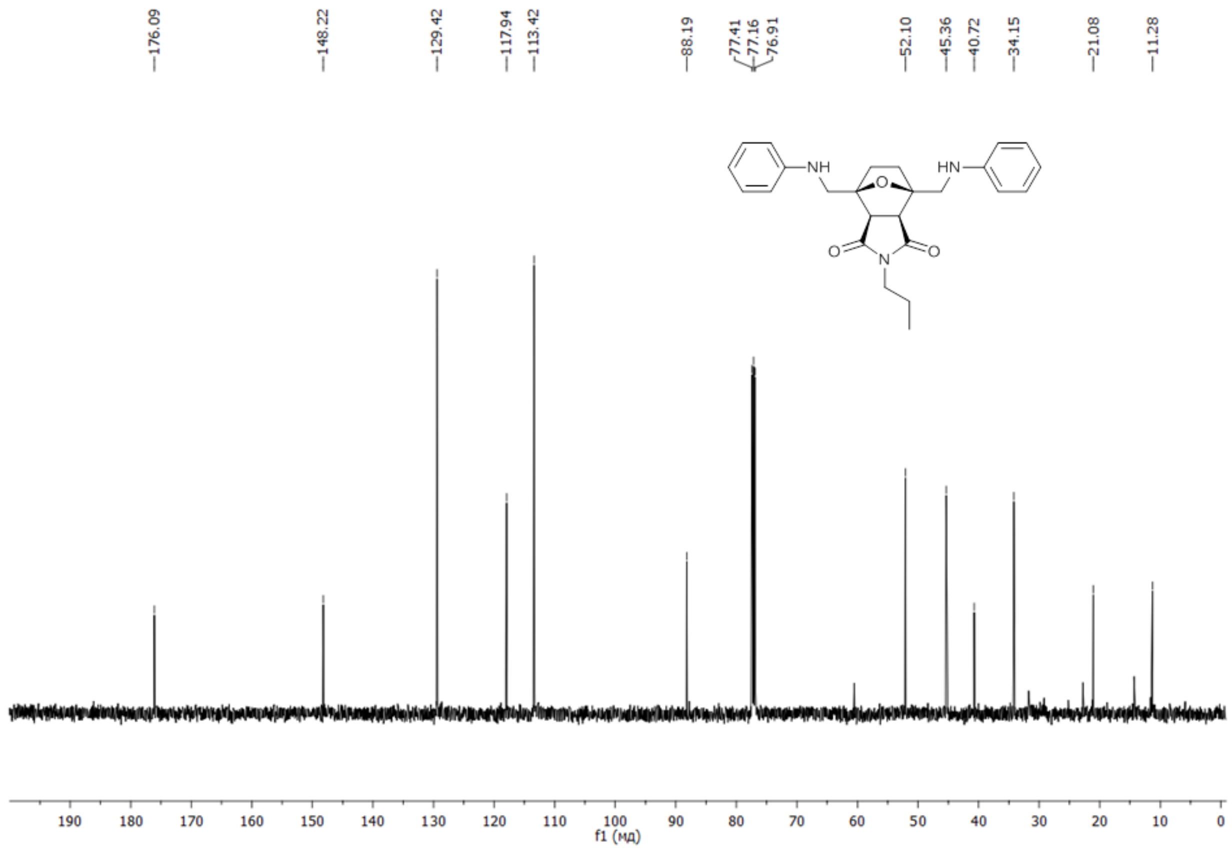

Fig. S14. ^{13}C NMR spectrum of compound 10-exo ($CDCl_3$, 298 K, 126 MHz).

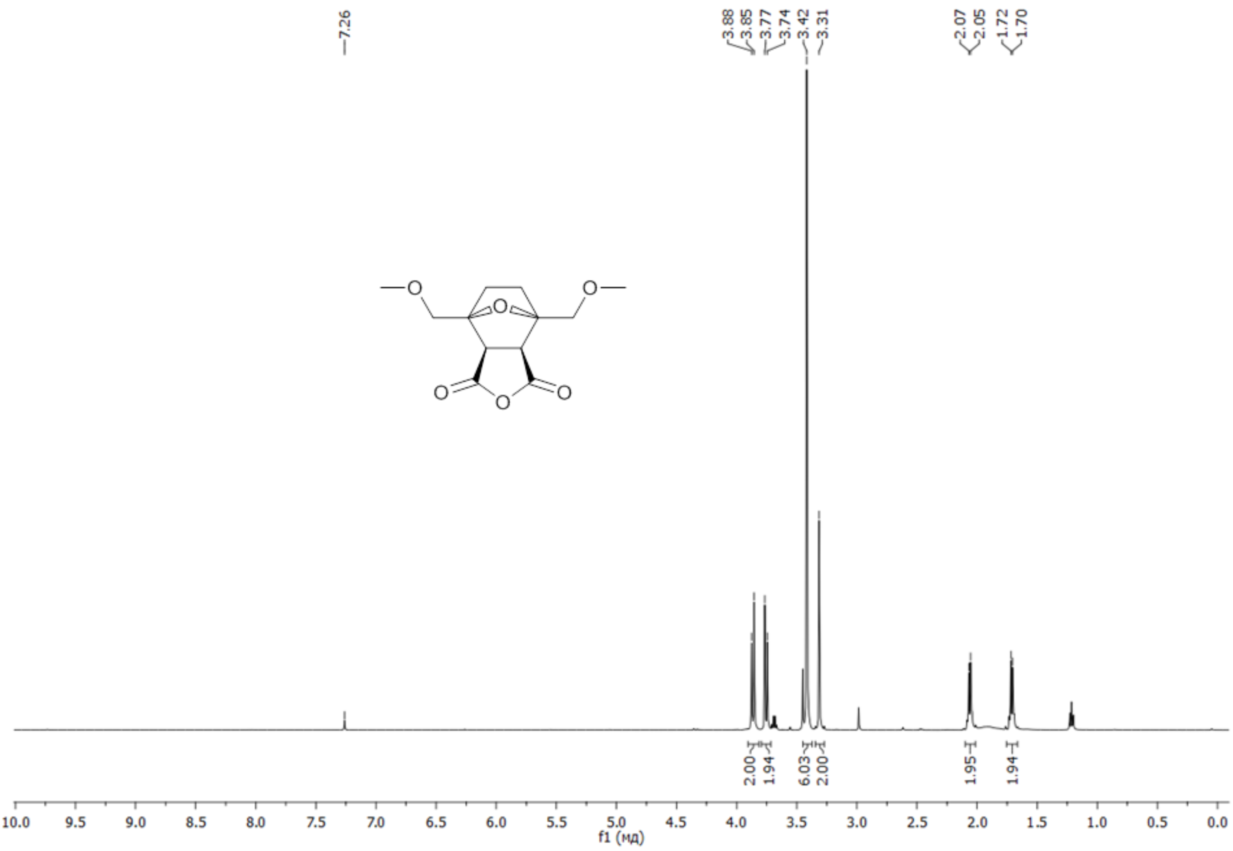

Fig. S15. ^{1}H NMR spectrum of compound 17 ($CDCl_3$, 298 K, 500 MHz).

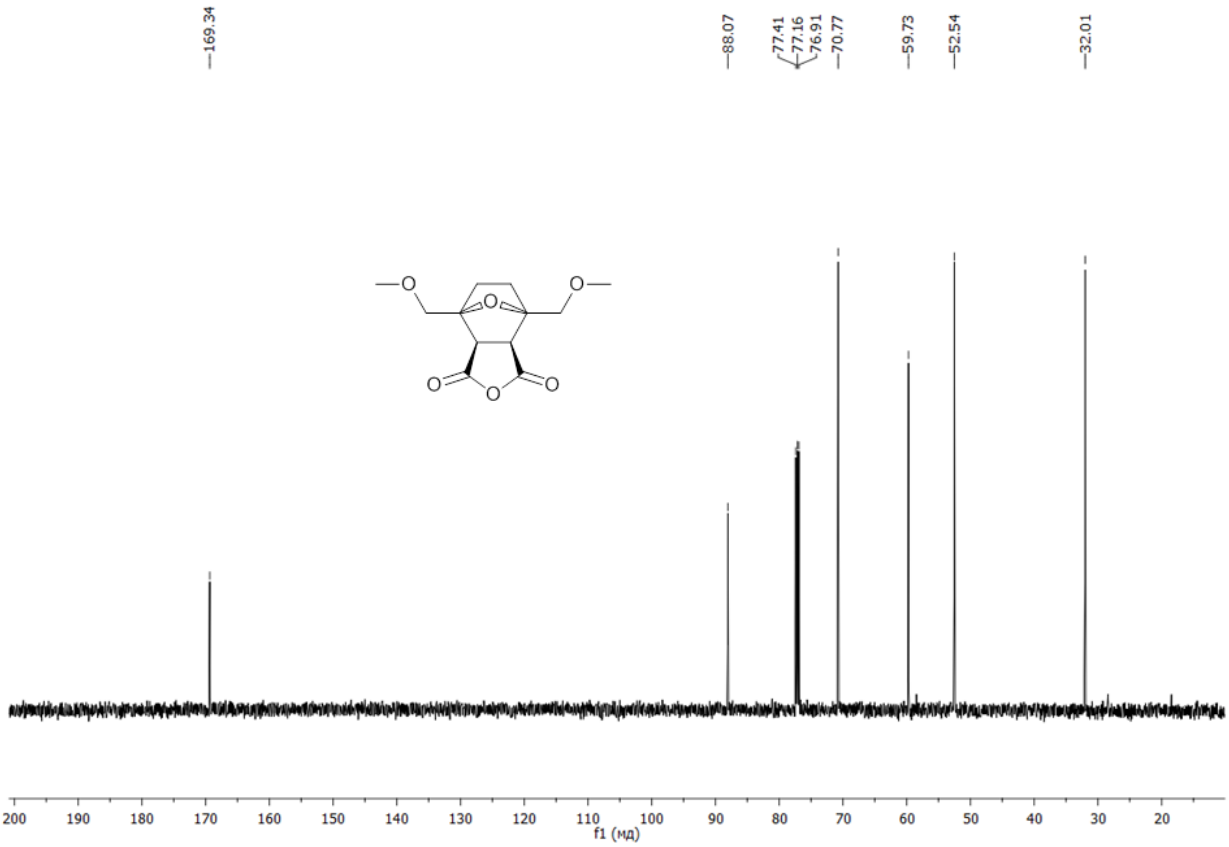

Fig. S16. ^{13}C NMR spectrum of compound 17 ($CDCl_3$, 298 K, 126 MHz).

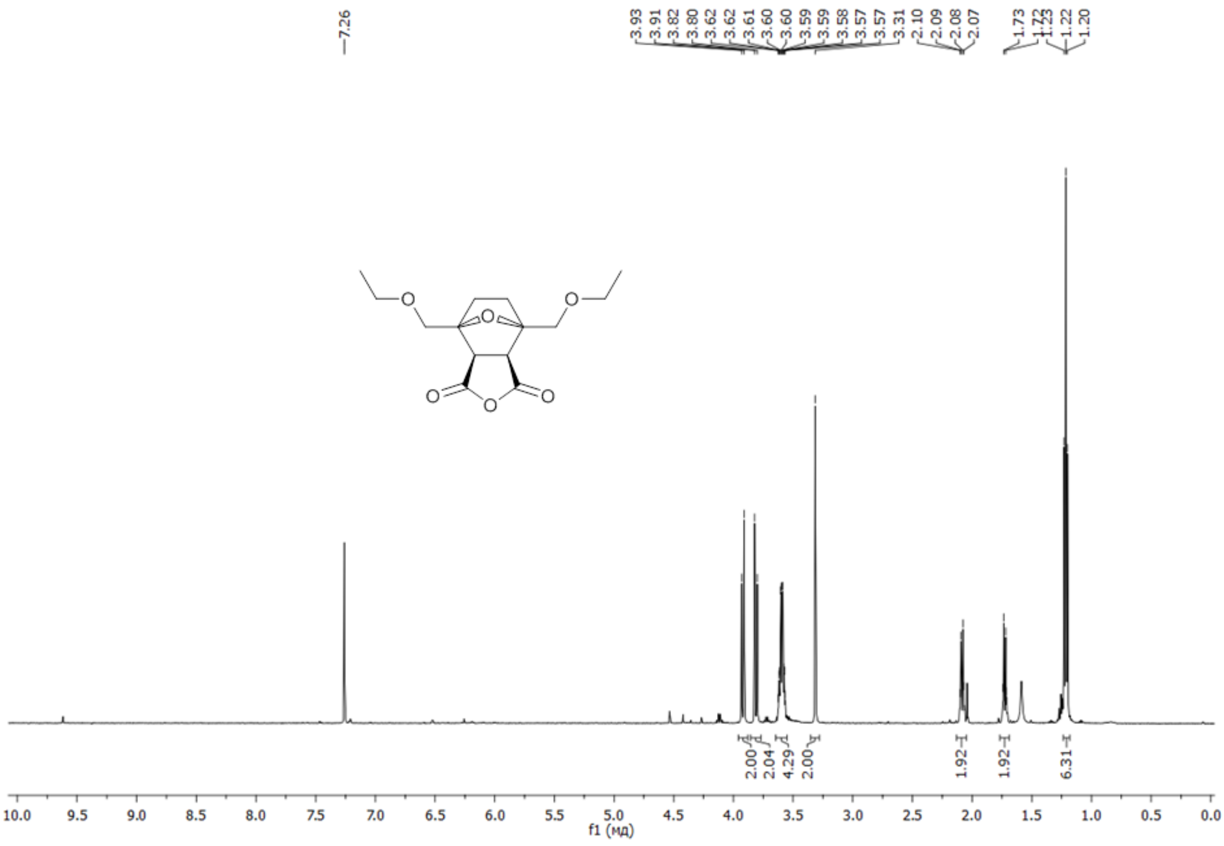

Fig. S17. [1]**H NMR spectrum of compound 18 ($CDCl_3$, 298 K, 500 MHz).**

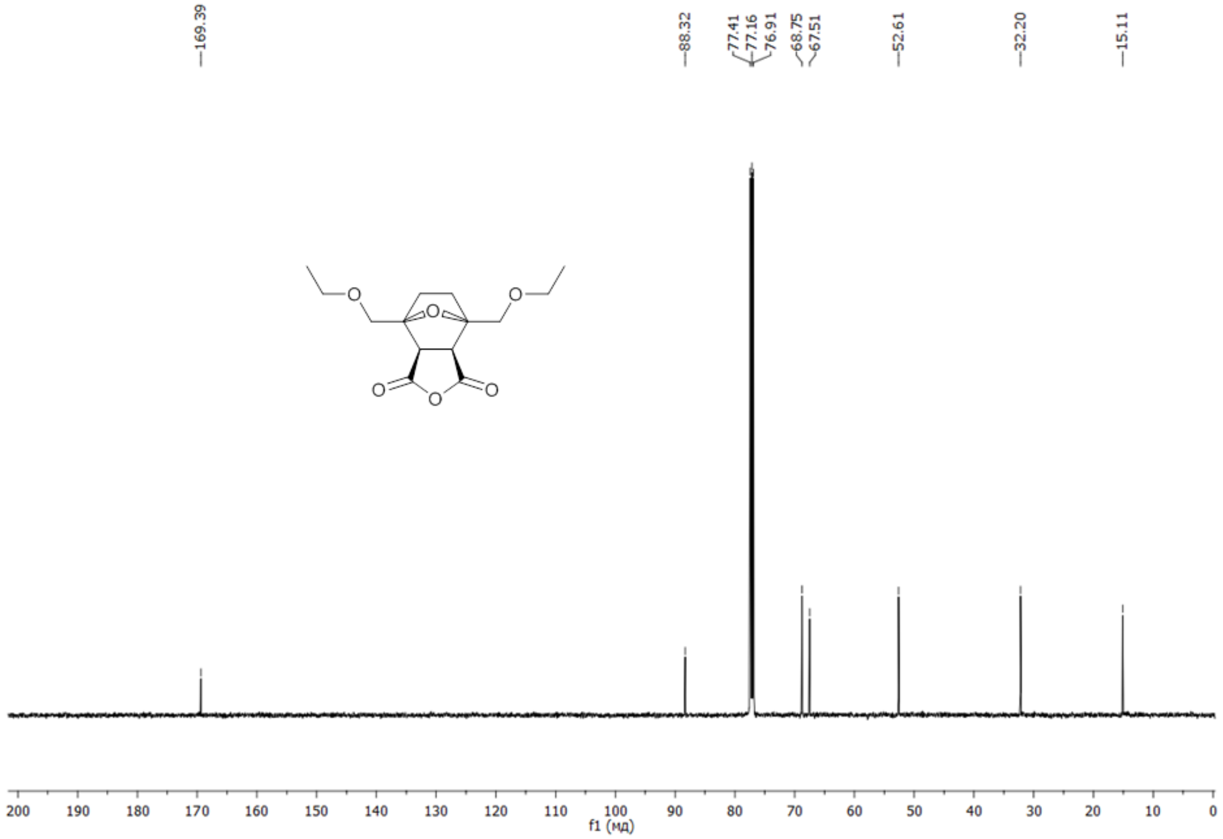

Fig. S18. [13]**C NMR spectrum of compound 18 ($CDCl_3$, 298 K, 126 MHz).**

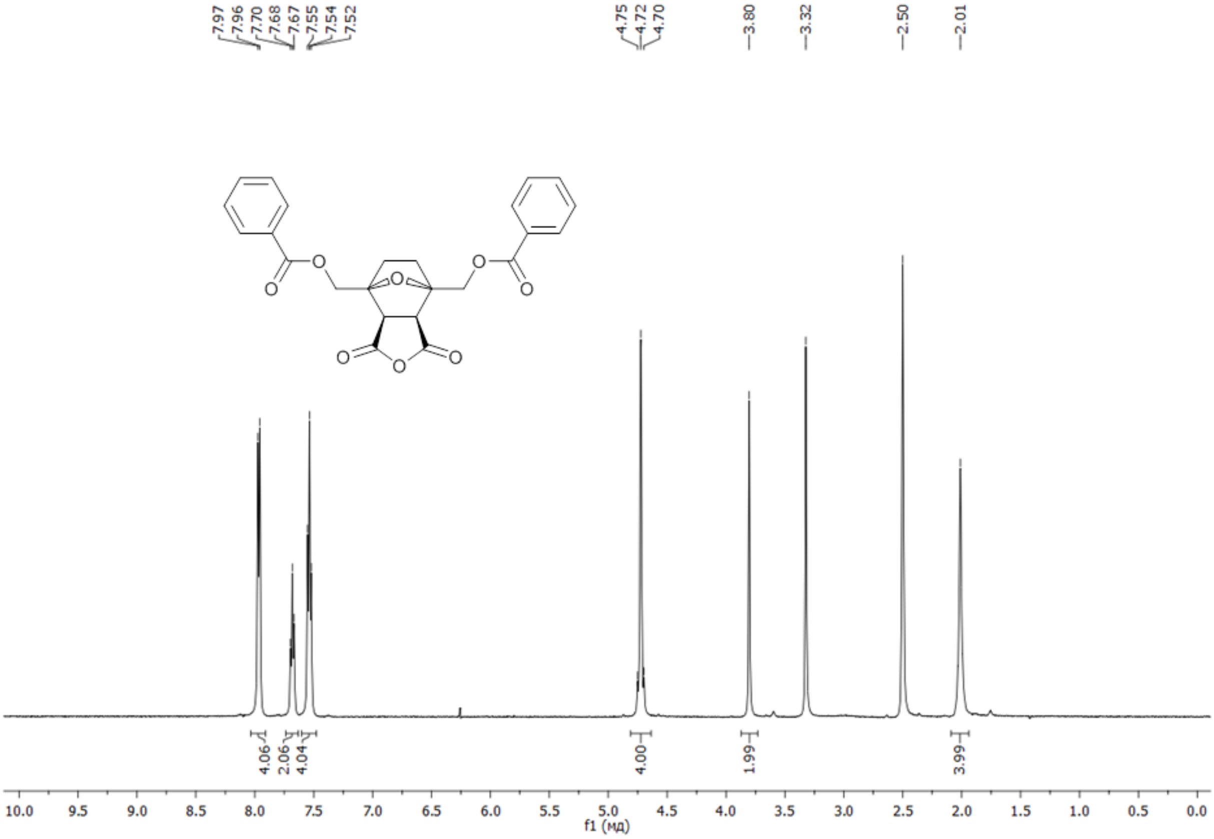

Fig. S19. [1]**H NMR spectrum of compound 19 (DMSO-*d*$_6$, 298 K, 500 MHz).**

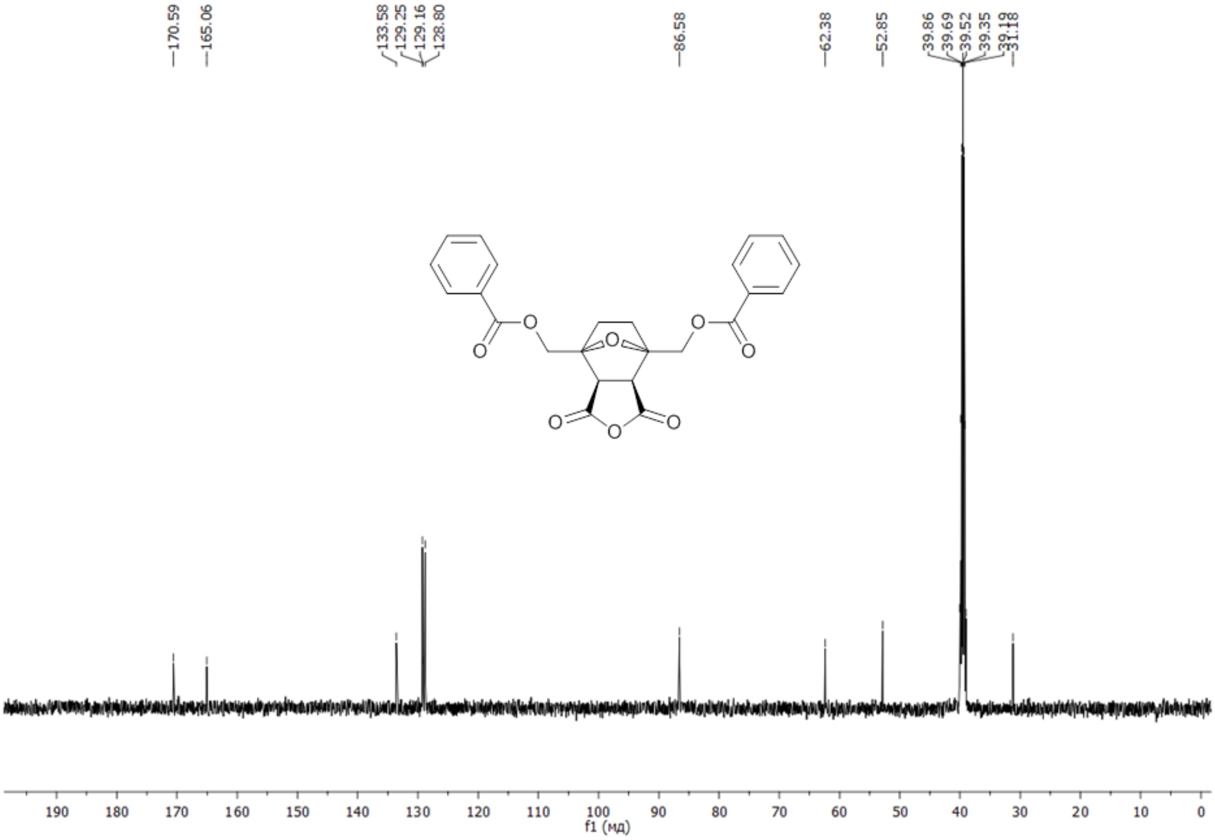

Fig. S20. [13]**C NMR spectrum of compound 19 (DMSO-*d*$_6$, 298 K, 126 MHz).**

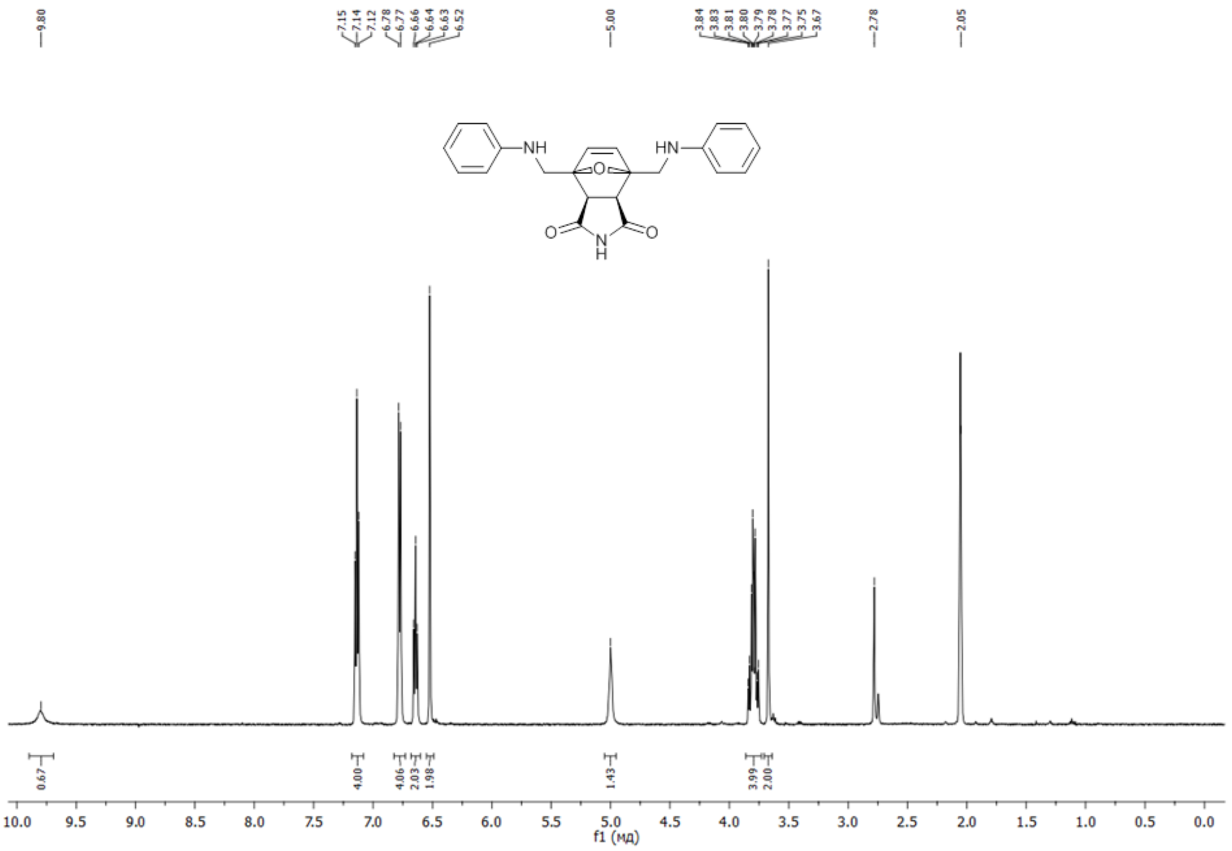

Fig. S21. ^{1}**H NMR spectrum of compound 9a (acetone-*d*$_6$, 298 K, 500 MHz).**

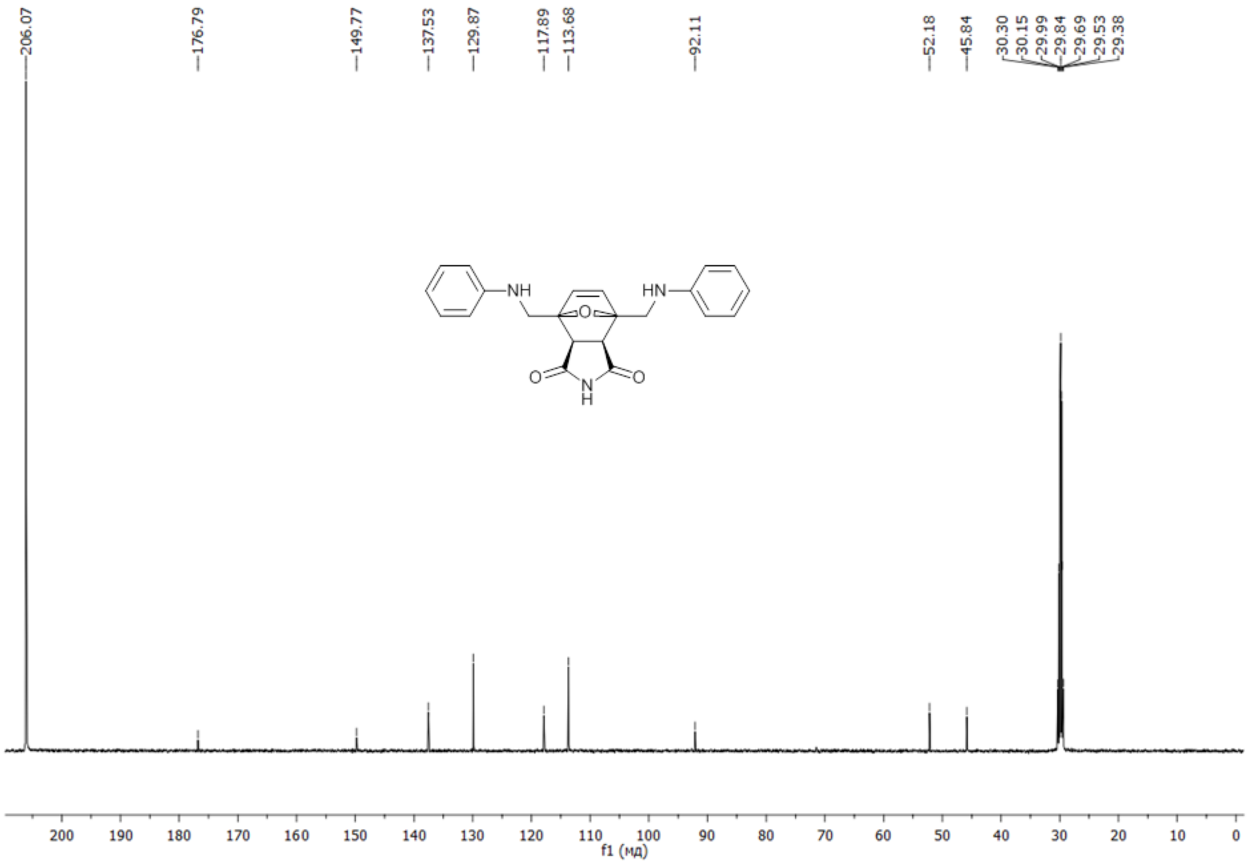

Fig. S22. ^{1}**H NMR spectrum of compound 9a (acetone-*d*$_6$, 298 K, 126 MHz).**

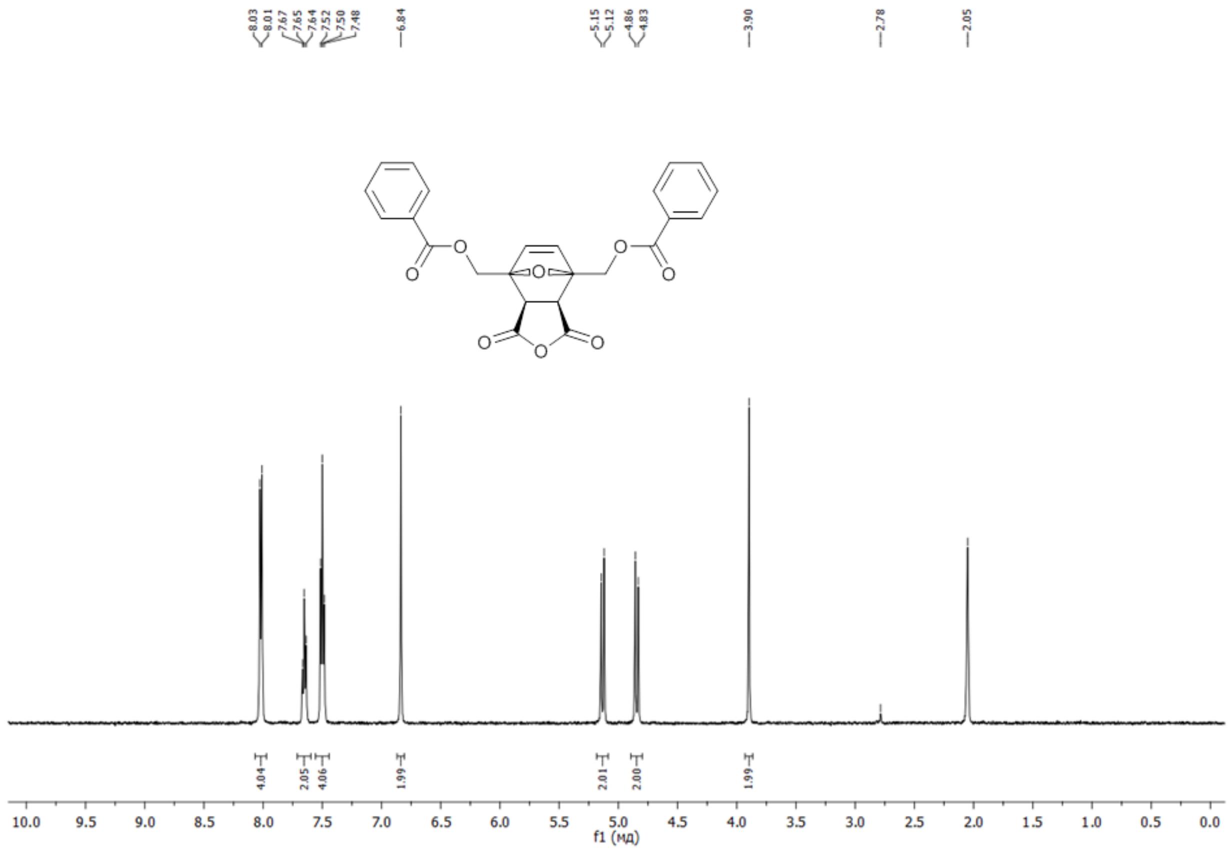

Fig. S23. ^{1}H NMR spectrum of compound 19a (acetone-d_6, 298 K, 500 MHz).

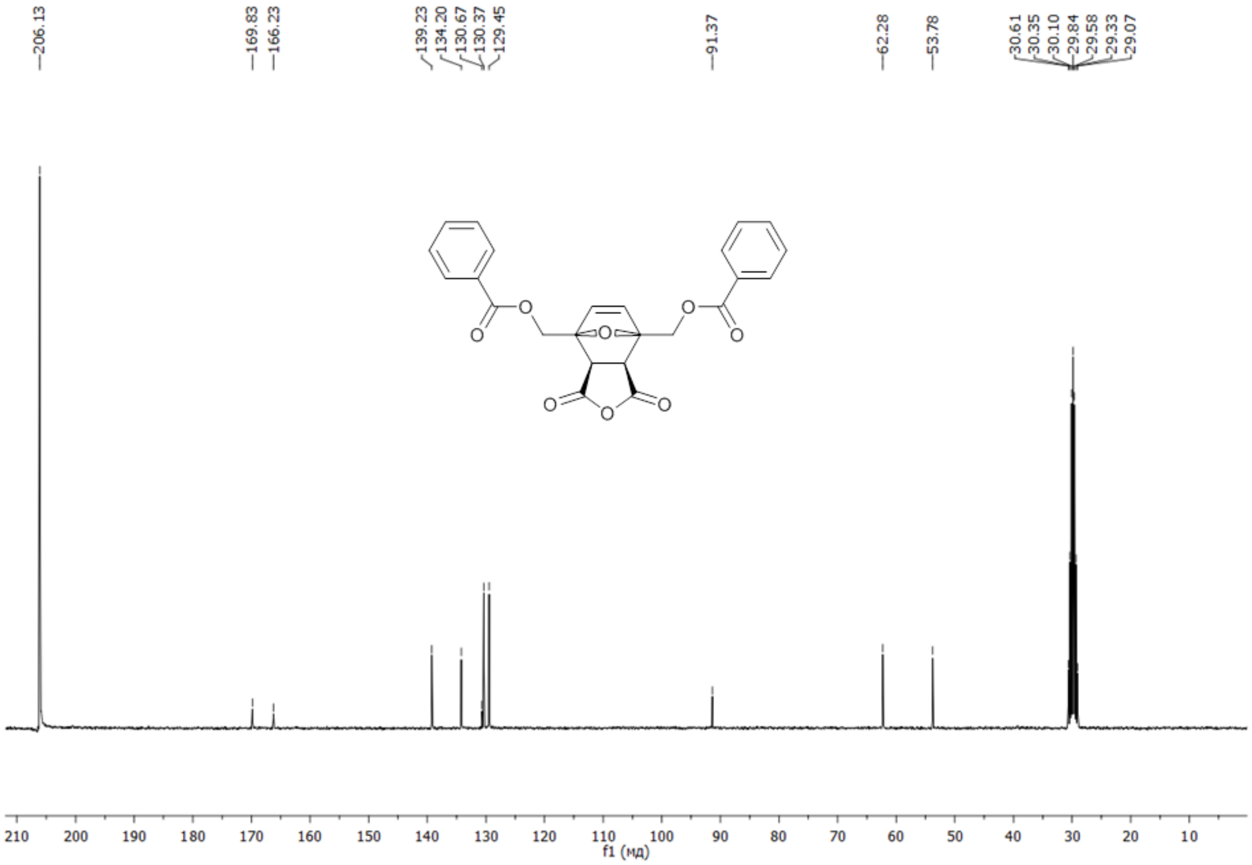

Fig. S24. ^{13}C NMR spectrum of compound 19a (acetone-d_6, 298 K, 75 MHz).

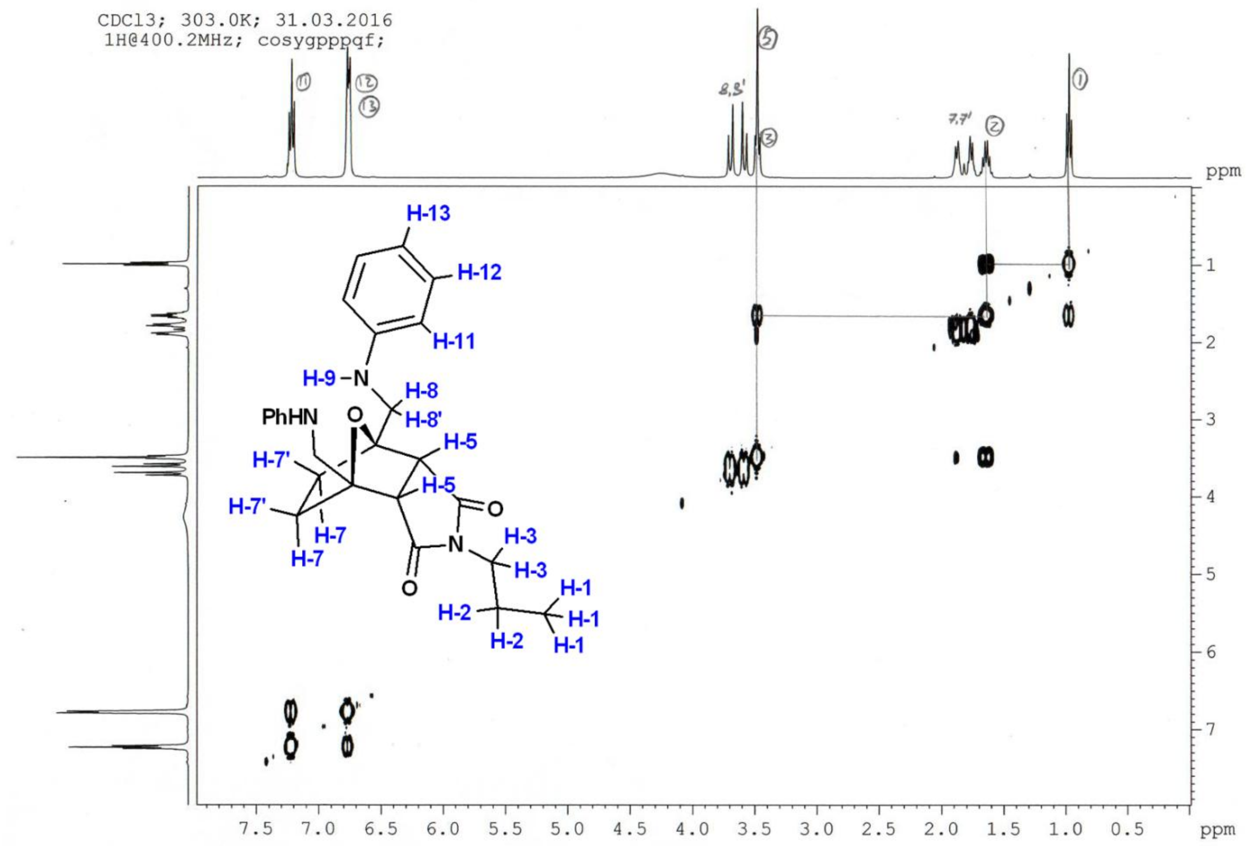

Fig. S25. **COSY spectrum of compound 10-endo ($CDCl_3$, 298 K, 400 MHz).**

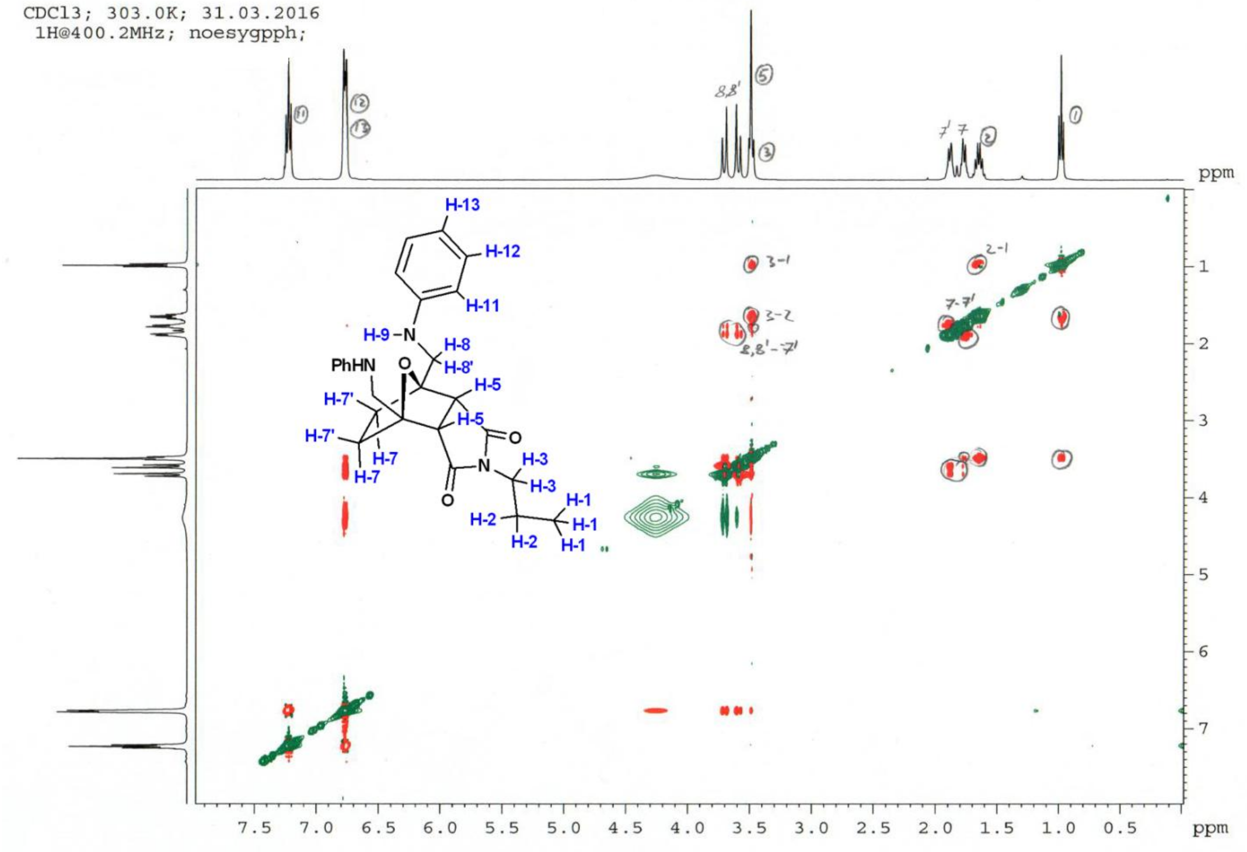

Fig. S26. **NOESY spectrum of compound 10-endo ($CDCl_3$, 303 K, 400 MHz).**

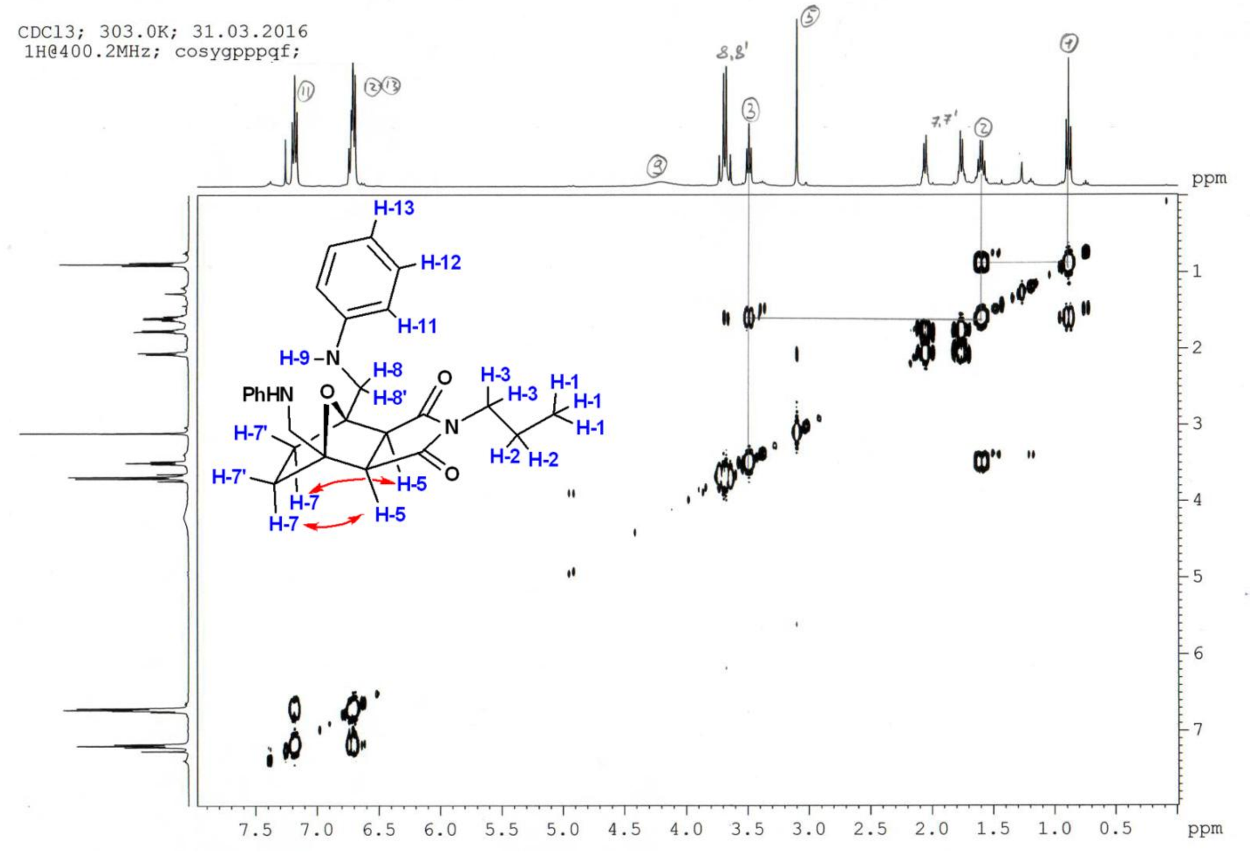

Fig. S27. COSY spectrum of compound 10-exo ($CDCl_3$, 303 K, 400 MHz).

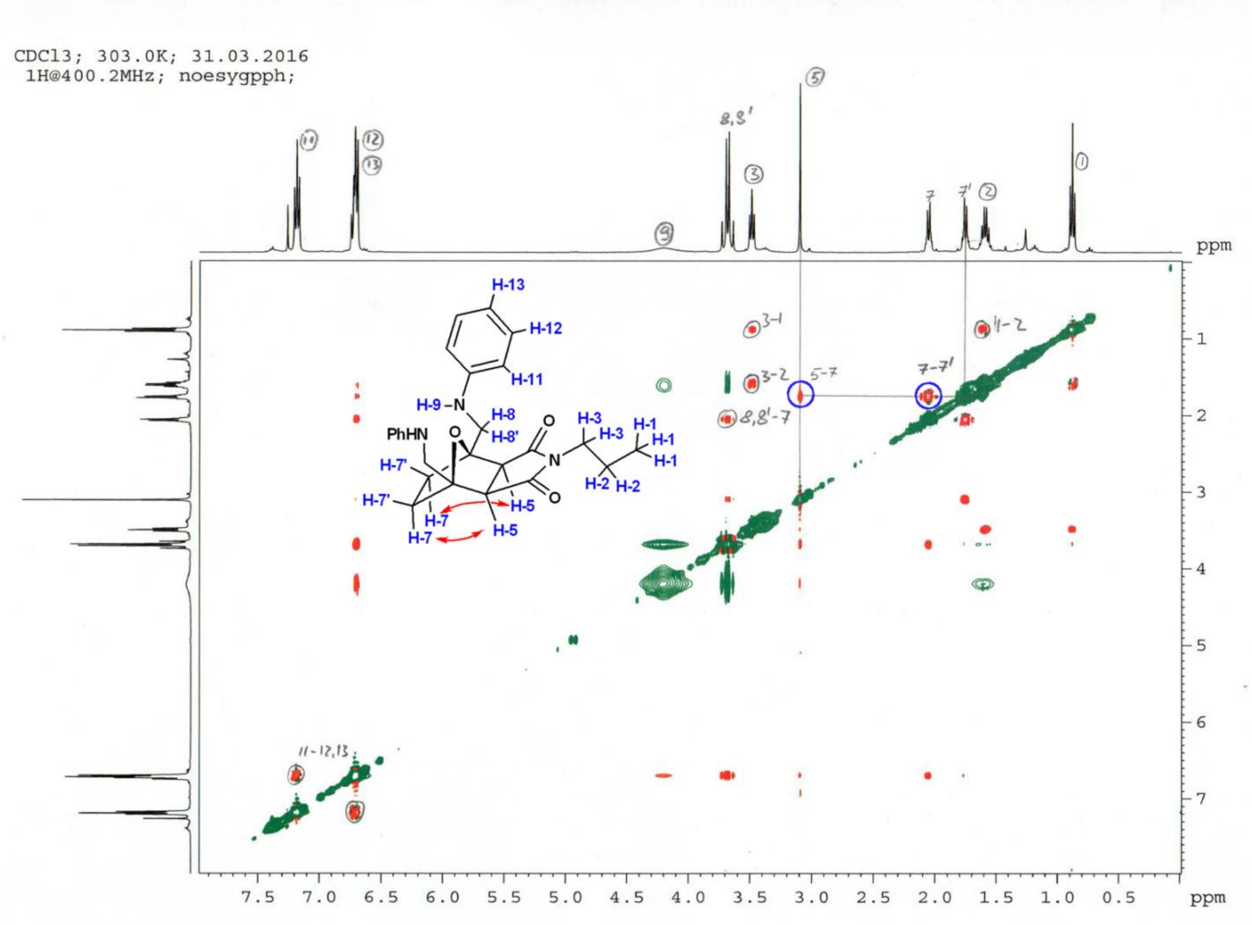

Fig. S28. NOESY spectrum of compound 10-exo ($CDCl_3$, 298 K, 400 MHz).

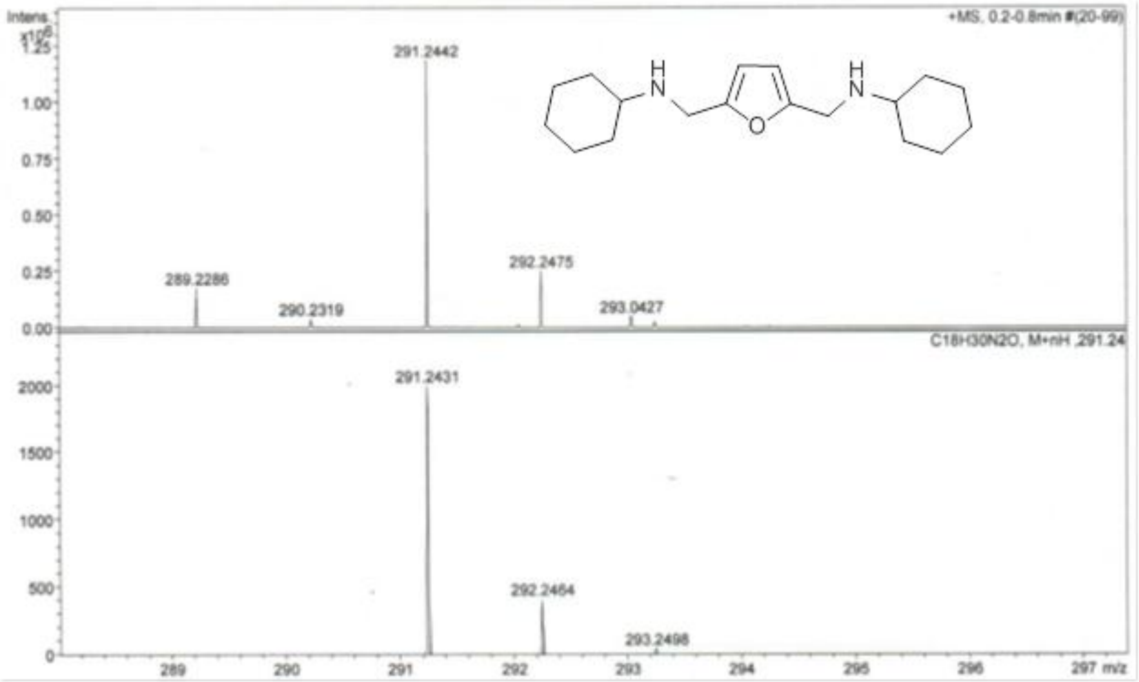

Fig. S29. HRMS (ESI) spectrum of compound 5.

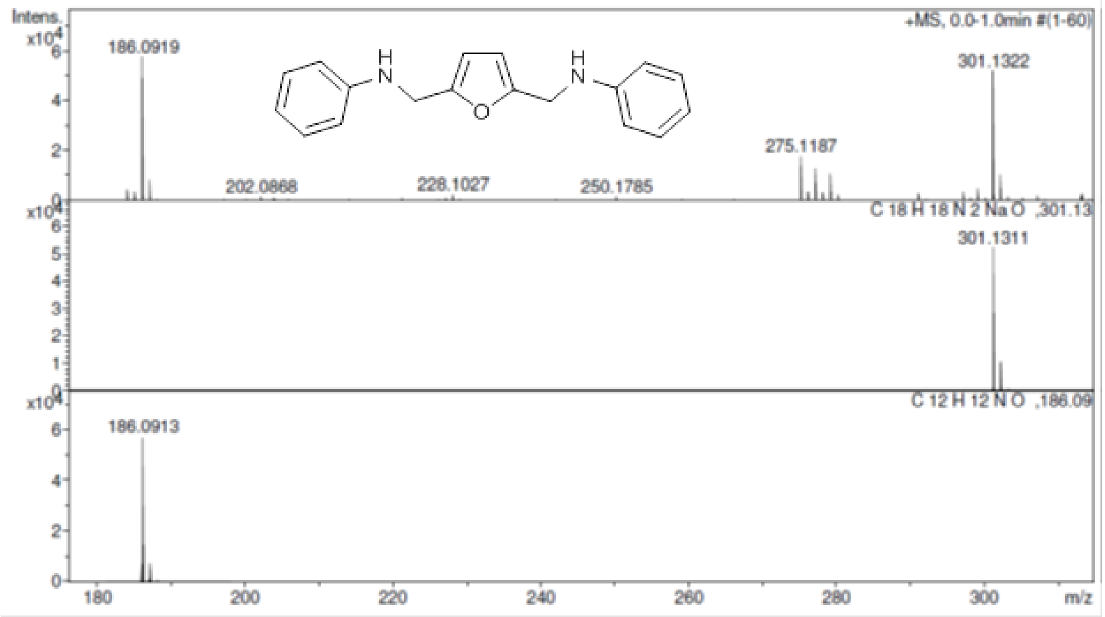

Fig. S30. HRMS (ESI) spectrum of compound 6.

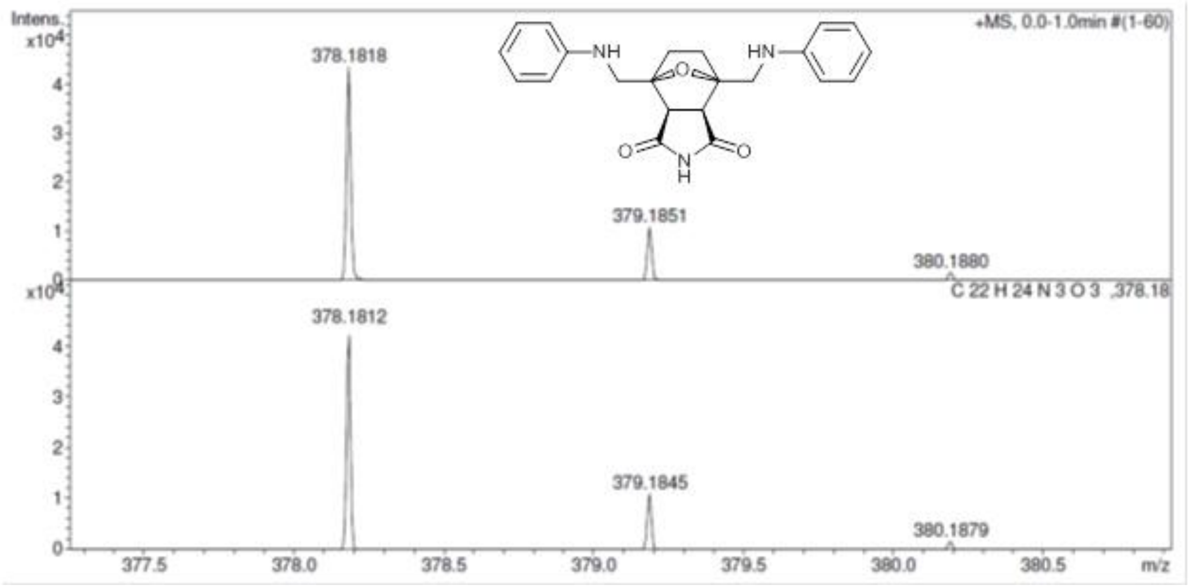

Fig. S31. HRMS (ESI) spectrum of compound 9.

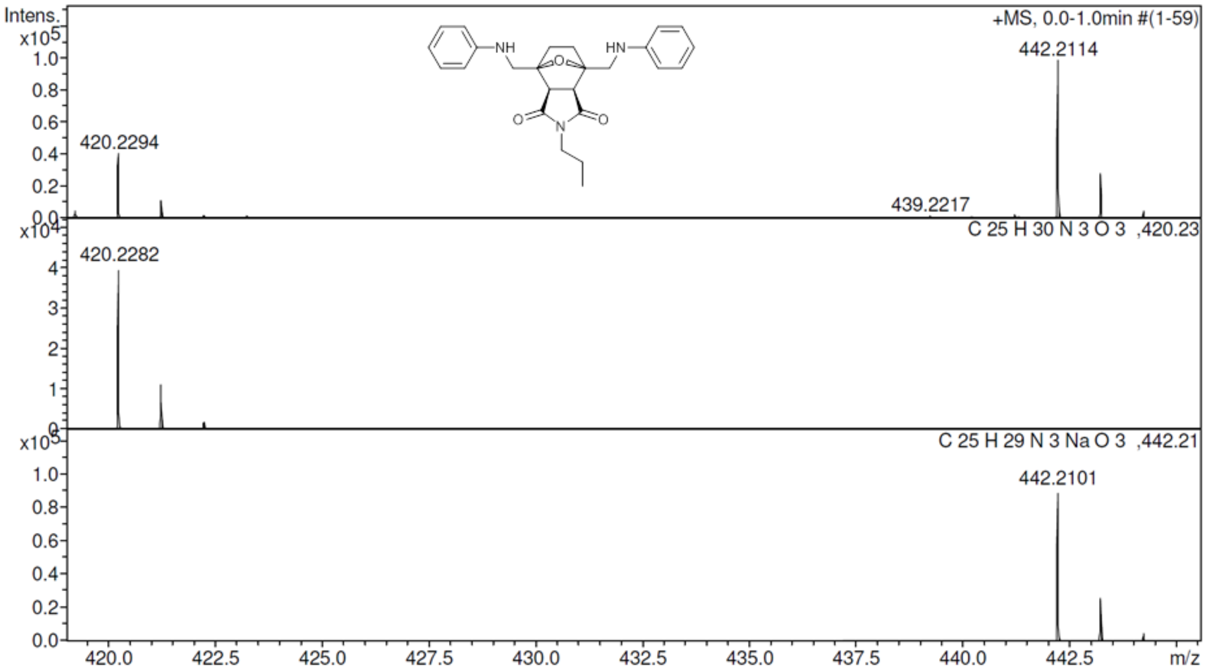

Fig. S32. HRMS (ESI) spectrum of compound 10-endo.

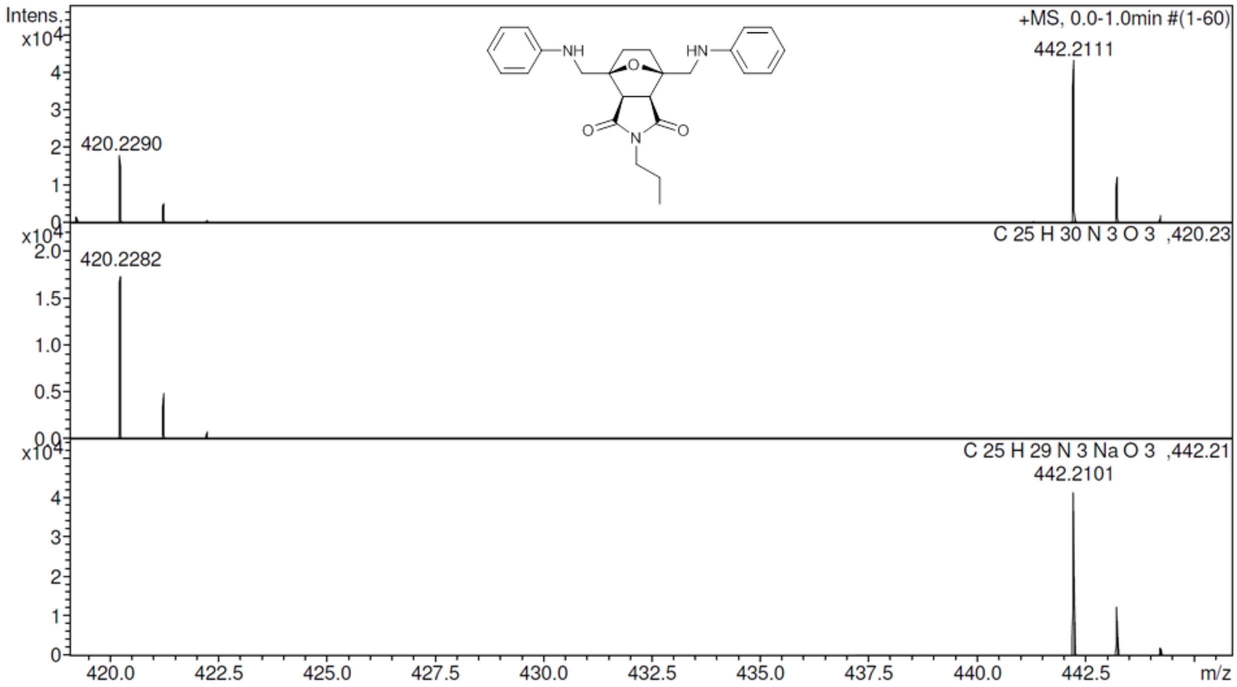

Fig. S33. HRMS (ESI) spectrum of compound 10-exo.

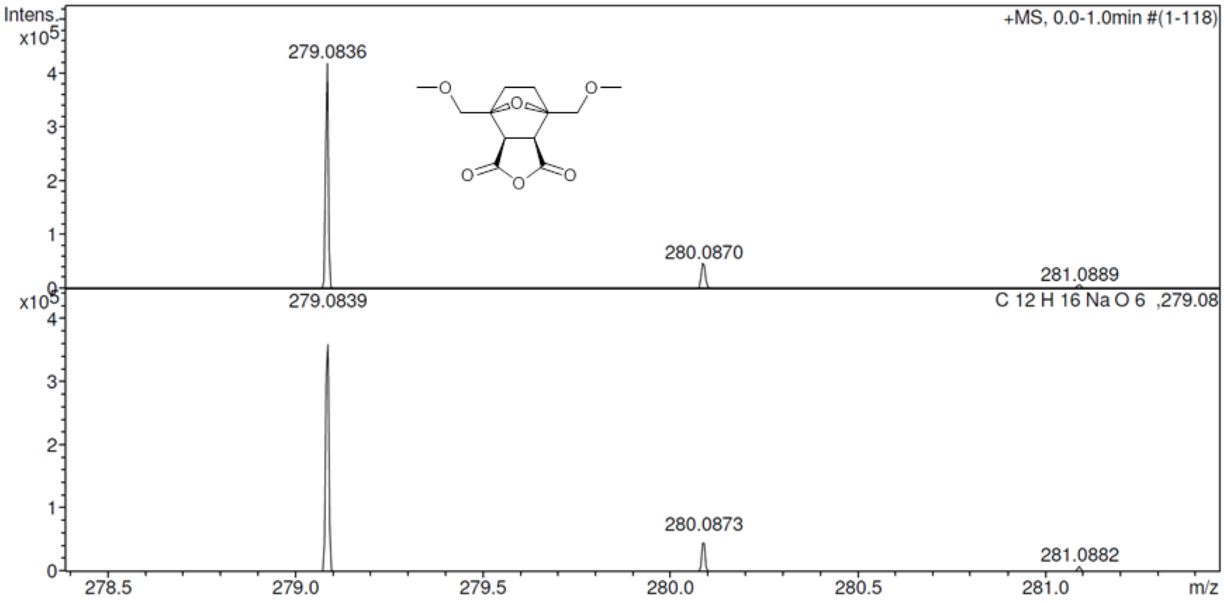

Fig. S34. HRMS (ESI) spectrum of compound 17.

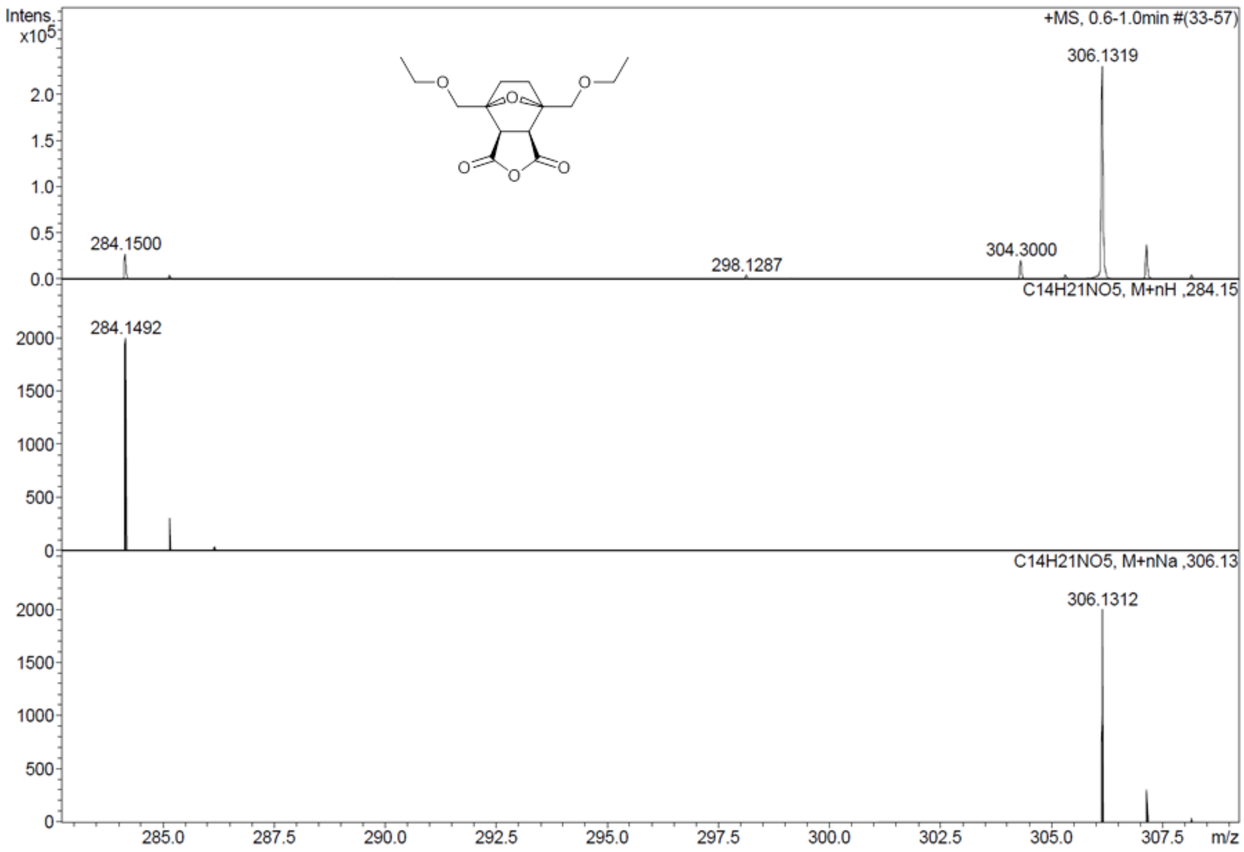

Fig. S35. HRMS (ESI) spectrum of compound 18.

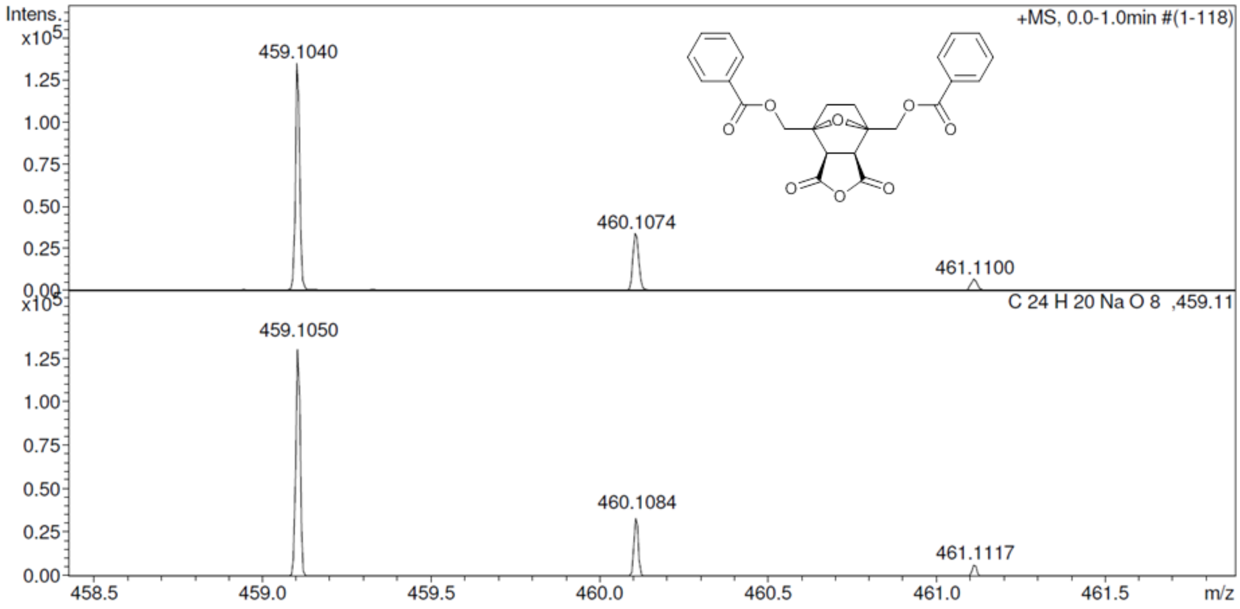

Fig. S36. HRMS (ESI) spectrum of compound 19.

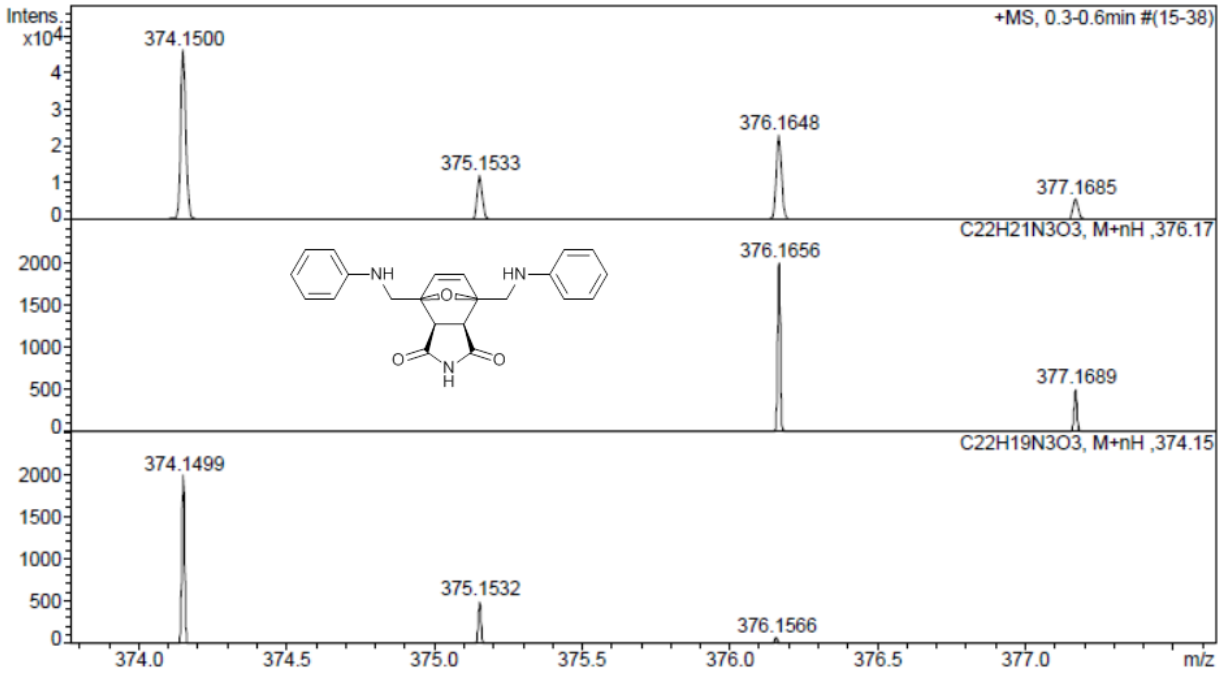

Fig. S37. HRMS (ESI) spectrum of compound 9a.

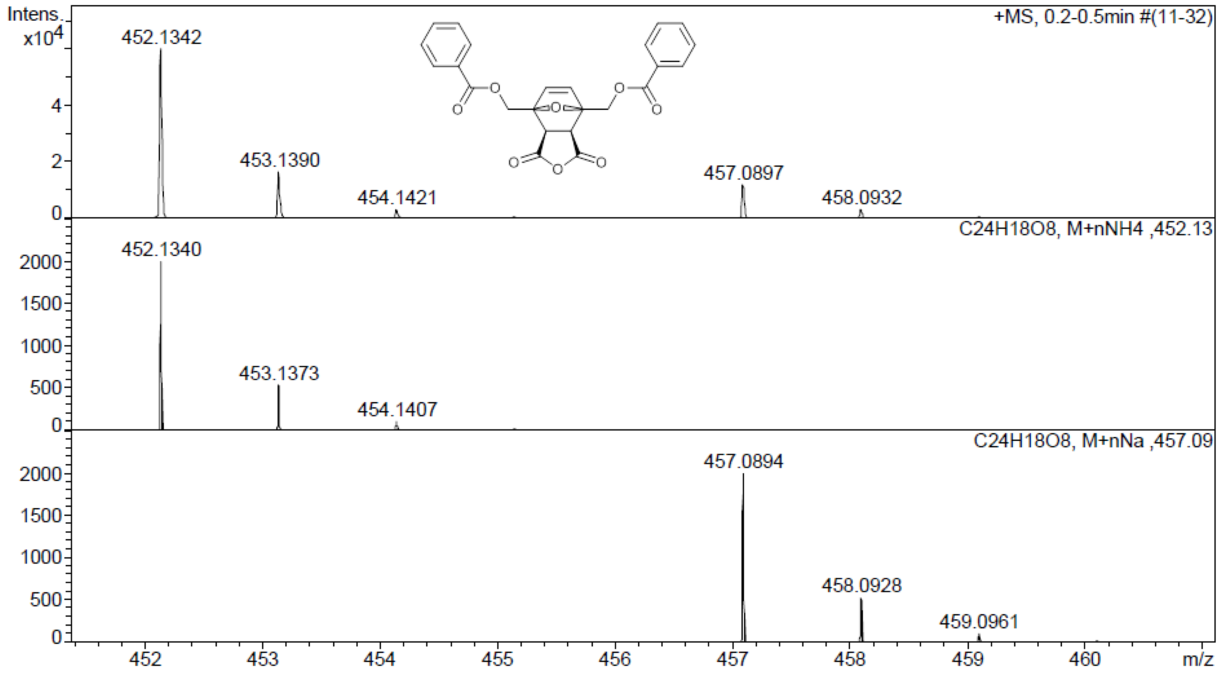

Fig. S38. HRMS (ESI) spectrum of compound 19a.

Preliminary studies of cytotoxicity of selected substances

Table S1. Cytotoxicity of selected substances towards HT-29 cells.

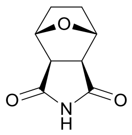

norcantharimide

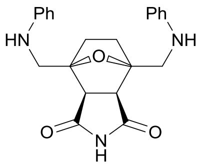

9

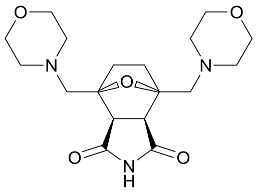

12

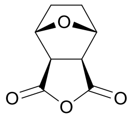

norcantharidin

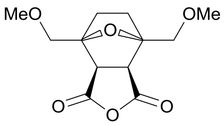

17

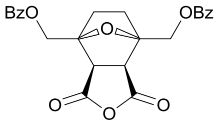

19

Entry	Substance	48-h IC_{50}, µM	72-h IC_{50}, µM
1	Norcantharidin	180	26
2	Norcantharimide	>5000	-[b]
3	**9**	350[a]	250
4	**12**	>5000	-[b]
5	**17**	>1000	-[b]
6	**19**	-[c]	-[c]

[a] - Did not kill all cells at the maximum concentration tested. [b] – Not tested due to low effect shown in 48h experiment. [c] - Hydrolysis of the product.
